# Identification of key differential genes in intimal hyperplasia induced by left carotid artery ligation

**DOI:** 10.7717/peerj.13436

**Published:** 2022-05-13

**Authors:** Lina Zhang, Jianjun Gu, Sichuan Wang, Fuming He, Kaizheng Gong

**Affiliations:** 1 Department of Cardiology, The Affiliated Hospital of Yangzhou University, Yangzhou University, Yangzhou, Jiangsu, China; 2 Department of Cardiology, Northern Jiangsu People’s Hospital, Yangzhou University, Yangzhou, Jiangsu, China

**Keywords:** Intimal hyperplasia, Transcriptome, KEGG pathway, Protein–protein interaction network

## Abstract

**Background:**

Intimal hyperplasia is a common pathological process of restenosis following angioplasty, atherosclerosis, pulmonary hypertension, vein graft stenosis, and other proliferative diseases. This study aims to screen for potential novel gene targets and mechanisms related to vascular intimal hyperplasia through an integrated microarray analysis of the Gene Expression Omnibus Database (GEO) database.

**Material and Methods:**

The gene expression profile of the GSE56143 dataset was downloaded from the Gene Expression Omnibus database. Functional enrichment analysis, protein-protein interaction (PPI) network analysis, and the transcription factor (TF)-target gene regulatory network were used to reveal the biological functions of differential genes (DEGs). Furthermore, the expression levels of the top 10 key DEGs were verified at the mRNA and protein level in the carotid artery 7 days after ligation.

**Results:**

A total of 373 DEGs (199 upregulated DEGs and 174 downregulated DEGs) were screened. These DEGs were significantly enriched in biological processes, including immune system process, cell adhesion, and several pathways, which were mainly associated with cell adhesion molecules and the regulation of the actin cytoskeleton. The top 10 key DEGs (Ptprc, Fn1, Tyrobp, Emr1, Itgb2, Itgax, CD44, Ctss, Ly86, and Aif1) acted as key genes in the PPI network. The verification of these key DEGs at the mRNA and protein levels was consistent with the results of the above-mentioned bioinformatics analysis.

**Conclusion:**

The present study identified key genes and pathways involved in intimal hyperplasia induced by carotid artery ligation. These results improved our understanding of the mechanisms underlying the development of intimal hyperplasia and provided candidate targets.

## Introduction

A growing body of evidence has shown that atherosclerosis is a chronic inflammatory process that narrows and hardens the arteries due to an excessive build-up of plaque in the tunica intima of the arterial wall (Daugherty, Tabas & Rader, 2015). Although percutaneous transluminal coronary angioplasty and drug-eluting stents are widely used in clinical treatment for coronary artery disease, they both inevitably trigger a high rate of in-stent restenosis (>10%) (Giacoppo et al., 2015). The major causes of in-stent restenosis are intimal hyperplasia and vascular remodeling (Liang et al., 2019). However, the pathogenesis of intimal hyperplasia is complex and not completed understood. Previous studies have suggested that angiogenesis, intimal formation, vascular remodeling, platelet aggregation, endothelial activation, inflammation, vascular smooth muscle cell (VSMC) proliferation, migration, and phenotypic transformation may be closely related to intimal hyperplasia (Chan et al., 2008; Esper et al., 2008; Mount et al., 2008). Therefore, elucidating the gene changes and mechanisms involved into intimal hyperplasia is vital to understand the process of neointimal hyperplasia and for developing new therapeutic strategies in the prevention of vascular restenosis.

Gene chip microarray technology is a recent development commonly used to detect changes in gene expression with high specificity and sensitivity. For example, Woods et al. (2002) identified that the tyrosine kinase receptor, EphB2, as a differentially expressed gene (DEGs), had an anti-proliferative effect in vascular smooth muscle cells (VSMCs) in response to continuous intravenous heparin administration in the rabbit model of arterial injury. Moreover, Hokamura et al. (2010) analyzed changes in the gene expression profiles of injured blood vessels of mice using DNA microarray assays. They demonstrated that bacteremia induced by *P. gingivalis* led to intimal hyperplasia associated with over-expressions of S100A9 and Smemb (Hokamura et al., 2010). Similarly, Stone et al. (2001) reported that the early and sustained under-expression of some proteasome genes may alter cell cycle control and matrix protein signaling, contributing to the unregulated proliferation of smooth muscle cells and the extracellular matrix in anastomotic intimal hyperplasia after prosthetic arterial grafting.

Herein, we downloaded and analyzed the original microarray dataset of intimal hyperplasia induced by the ligation of the left carotid artery from the Gene Expression Omnibus (GEO) (*i.e*. GSE56143) to obtain potential DEGs related to intimal hyperplasia. In addition, their functions and pathways were analyzed using the Gene Ontology (GO) and Kyoto Encyclopedia of Genes and Genomes (KEGG) pathway enrichment analyses, respectively. The major key genes related to intimal hyperplasia were identified and the transcriptional regulation based on DEGs transcription factors (TFs) was predicted using TRRUST2.0 software. Lastly, we established a mouse model of vascular intimal hyperplasia by ligating the left carotid artery and verified the changes of the key genes at the mRNA and protein levels.

## Methods

### Microarray data

The microarray expression dataset (GES56143), including three left carotid artery (LCA) and three right carotid artery (RCA) tissue samples was downloaded from the GEO database (https://www.ncbi.nlm.nih.gov/geo/). The data were based on the GPL6887 platforms (Illumina MouseWG-6 v2.0 Array; Georgia Tech & Emory University, San Diego, CA, USA).

### Identification of DEGs

The GEO2R online analysis tool was used to identify DEGs. Differential expression analysis of LCA *vs*. RCA was performed on the samples using the classical Bayesian test in the limma package. All reported *P*-values of DEGs were determined by log2FC > 1 or log2FC < −1 (FC, fold change) and values of <0.05 were regarded as statistically significant.

### GO terms and pathway enrichment analysis

The functions and related pathways of DEGs were explored using the Database for Annotation, Visualization and Integrated Discovery (DAVID 6.8), which includes KEGG pathway and Gene Ontology (GO) analysis (containing three subsets of OTERM_BP_DIRECT, GOTERM_CC_DIRECT, and GOTERM_MF_ DIRE CT). A cut-off *P*-value <0.05 was defined as statistically significant.

### Integration of the protein–protein interaction (PPI) network and identification of key genes

The PPI network was constructed based on the DEG-encoded proteins and the functional interactions were analyzed using the STRING online tool (http://string-db.org) (Szklarczyk et al., 2017). The interactive network was then introduced into Cytoscape (Doncheva et al., 2012), where genes with the marginal degree ≥48 were defined as key genes.

### TFs analysis

The TRRUST2.0 online tool (https://www.grnpedia.org/trrust/) was used to predict the transcription factor based on DEGs. The significant TF and possibly regulated genes were identified using the model of multiple parameters analysis. The output results were visualized through Cytoscape software.

### Animals

The mice used in this study were male (*n* = 18), specific pathogen-free (SPF) and 8–12 weeks old C57BL/6, and weight 20–25 g. They were obtained from the Comparative Medical Center of Yangzhou University (Yangzhou, China). The mice were kept at a constant temperature (24 ± 1 °C), humidity (60 ± 5%), 12 h light/dark cycle (6:00 am to 6:00 pm), and were fed a standard mouse pellet diet *ad libitum*. All protocols in this study were approved by the Institutional Animal Care and Use Committee of the Affiliated Hospital of Yangzhou University (2018-YKL11-27-016), and were consistent with the Guide for the Care and Use of Laboratory Animals published by the US National Institutes of Health (National Research Council (US) Committee for the Update of the Guide for the Care and Use of Laboratory Animals, 2011).

### Carotid artery ligation model

In order to exclude the effect of estrogen on vascular damage, only adult male mice were used in this experimental study after a 7-day acclimatization period to the preoperative environment. Briefly, there were two groups in this study, the left carotid arteries (LCA) were the ligation group, and the contralateral right carotid artery received a sham-operation to serve as the intra-animal control (RCA). For carotid artery ligation, ketamine (80 mg/kg intraperitoneal) and xylazine (5 mg/kg intraperitoneal) were combined to anesthetize mice and the LCA was exposed through a midline cervical incision and ligated with an 5–0 silk suture just proximal to the bifurcation, as described previously (Xing et al., 2008). A similar procedure was performed but without ligation on the right carotid artery. All experimental mice were euthanized with anesthetic on the 7th day after carotid artery ligation. Then, the mice were processed for morphological and biochemical studies at specific time points after surgery.

### Histological analysis

The carotid artery tissues of the mice were fixed with 4% paraformaldehyde, dehydrated, and embedded in paraffin for tissue section after ligation of the carotid artery for 7 days. A continuous tissue section 5 μm thick was taken from the cross-section of carotid artery, 500 μm from the ligation point. The histological images of the cross-sections from the center of the injured segment were stained by Weigert (Yuanye Bio-Technology, Shanghai, China). The intimal areas, medial areas and the intima to-media (I/M) ratios were measured using Image Pro Plus software (version 6.0; Media Cybernetics, Rockville, MD, USA) to assess the extent of the injury response and these samples were analyzed using GraphPad Prism 7.

### RTqPCR

The mRNA expression levels of the DEGs were measured using real-time qPCR, the primers were designed through the Pubmed website and were synthesized by the Shanghai Shenggong Company of China. The vascular tissues were collected 7 days after carotid artery ligation (*n* = 6) and were extracted with TRIzol reagent (Tiangen, Beijing, China) to obtain the total mRNA. This was then reverse-transcribed into cDNA using the PrimeScript™ RT regent kit with gDNA Eraser (Takara No. RR047A; Takara Bio Inc., Shiga, Japan). The synthesized cDNA was amplified by real-time quantitative PCR analysis using TB Green Premix Ex Taq™II (Takara No. RR820A; Takara Bio Inc., Shiga, Japan) in CFX96 Real-Time System (BioRad, Hercules, CA, USA). The levels of target gene mRNAs were normalized using GAPDH mRNA and were then standardized to the mRNA level of the RCA group. These data were further analyzed using GraphPad Prism 7. The forward and reverse primers pairs used for RTqPCR are shown in Table S1.

### Immunohistochemistry

To further validate the findings for DEGs expression at the protein level in the ligated vessels, standard immunohistochemical techniques were used to detect the top 10 DEGs in paraffin-embedded sections of the carotid artery. In brief, deparaffinized and hydrated sections were quenched with 3% hydrogen peroxide, digested with compound digestive juice, and then blocked with 5% bovine serum albumin. Tissues were then incubated at 4 °C overnight with the primary antibody. Then, anti-Ptprc (20103-1-AP; Proteintech, Rosemont, IL, USA), anti-Tyrobp (Santa Cruz, sc-166084), anti-Emr1 (Santa Cruz, sc-365340), anti-Itgb2 (10554-1-AP; Proteintech, Rosemont, IL, USA), anti-Itgax (GB11059; Servicebio, Wuhan, China), anti-Ctss (Santa Cruz, sc-271619), anti-Ly86 (Santa Cruz, sc-390613), anti-CD44 (GB112054; Servicebio, Wuhan, China), anti-Aif1 (GB11105; Servicebio, Wuhan, China), and anti-Fn1 (Santa Cruz, sc-18825) were diluted at 1/200. A streptavidinebiotin complex kit (Wuhan Booster Biological Technology, Wuhan, China) was used for subsequent steps, according to the manufacturer’s instructions. Chromogenic development was accomplished by diaminobenzidine–hydrogen peroxide. Slides were slightly counterstained with hematoxylin and dehydrated, and then coverslips were applied. Positive cells was determined using digital imaging at 100× magnification, and all positive cells counts were generated from three sections of each artery sample, as evaluated by an investigator who was blinded to the treatment protocols.

### Western blotting analysis

Protein samples were extracted from carotid arteries with a lysis buffer. The protein concentration was determined by the BCA method (Beyotime Institute of Biotechnology, Jiangsu, China). All samples were separated with different concentrations of SDS–PAGE according to different molecular weights, and transferred to an Immun-Blot polyvinylidene difluoride membrane (Bio-Rad, Hercules, CA, USA), membranes were blocked in 5% BSA (Solarbio, Beijing, China) in 1xTBST and were then incubated with anti-β-Actin (CTS, 8H10D10), anti-Ptprc (20103-1-AP; Proteintech, Rosemont, IL, USA), anti-Itgb2 (10554-1-AP; Proteintech, Rosemont, IL, USA), anti-Itgax (GB11059; Servicebio, Wuhan, China), anti-Ctss (Santa Cruz, sc-271619), anti-Ly86 (Santa Cruz, sc-390613), anti-CD44 (GB112054; Servicebio, Wuhan, China), anti-Aif1 (Santa Cruz, sc-32725) primary antibodies with working concentration about 1 μg/mL, and a horseradish peroxidase–conjugated secondary antibody, respectively. The bands were visualized using the Super Western Sensitivity Chemiluminescence Detection System (Thermo Fisher, Waltham, MA, USA). Autoradiographs were quantified by densitometry (NIH Image J).

### Statistical analysis

Data results were expressed as mean ± SEM in GraphPad Prism 7 (GraphPad Software Inc., San Diego, CA, USA). A two-side, unpaired Student’s *t*-test was used to analyze the difference between the two groups of data with normally distributed variables., and a *P* < 0.05 was considered to be statistically significant.

## Results

### Identification and GO enrichment analysis for DEGs

**Figure 1:**
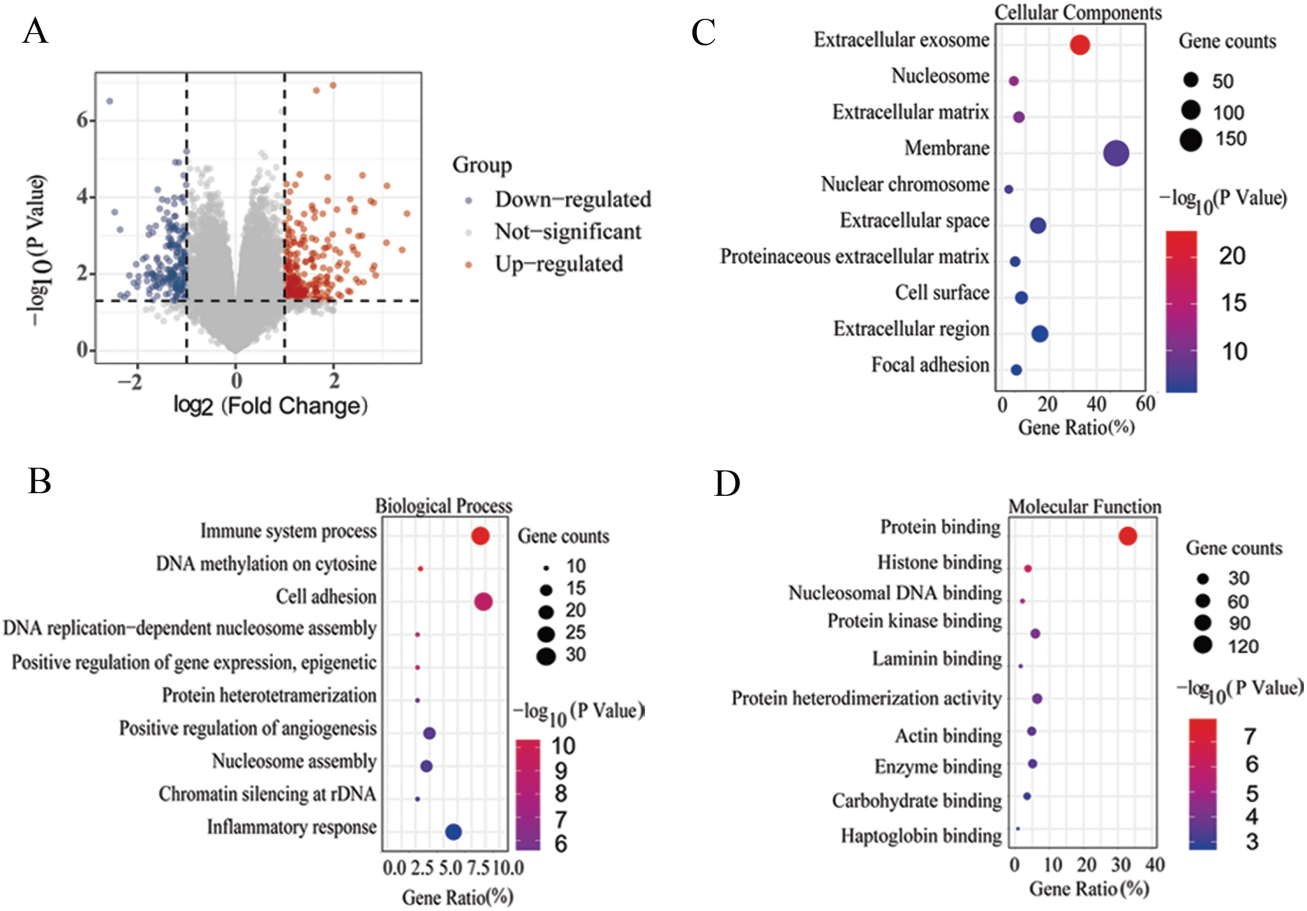
Differentially expressed genes (DEGs) on the volcano plot and GO enrichment analysis of common DEGs. (A) The DEGs of LCA *vs*. RCA on the volcano plot. The abscissa represents the difference in the fold change of gene expression in different groups, and the ordinate represents the *P* value of the expression difference. Gray dots represent unchanged genes. Red dots represent up-regulated genes, and blue dots represent down-regulated genes. GO enrichment analysis of common DEGs. GO analysis classified the DEGs into three groups, showing the top 10. (B) Biological processes. (C) Cellular component. (D) Molecular function. Gene ratio: The gene ratio was defined as the ratio of the number of differential genes annotated in the GO term to the total number of differential genes.

We used the GEO2R tool to analyze the DEGs in order to obtain the changes of the vascular transcriptome in the intimal hyperplasia model induced by carotid artery ligation. As shown in Fig. 1A and Table S2, a total of 373 DEGs were identified in the LCA/RCA group, including 199 upregulated genes and 174 downregulated genes.

Next, to understand the biological function of DEGs, the enrichment results were obtained through GO using DAVID version 6.8. The DEGs mainly enriched several biological processes, including immune system process, DNA methylation on cytosine, cell adhesion, DNA replication-dependent nucleosome assembly, positive regulation of gene expression, epigenetics, protein heterotetramerization, positive regulation of angiogenesis, nucleosome assembly, chromatin silencing at ribosome DNA (rDNA), and inflammatory response (Fig. 1B and Table S3). DEGs were mainly enriched in the extracellular exosome, nucleosome, extracellular matrix, membrane, nuclear chromosome, extracellular space, proteinaceous extracellular matrix, cell surface, extracellular region, and focal adhesion for cellular components (Fig. 1C and Table S4). Molecular function analysis showed that DEGs were mainly enriched in protein binding, histone binding, nucleosomal DNA binding, protein kinase binding, laminin binding, protein heterodimerization activity, actin binding, enzyme binding, carbohydrate binding, and haptoglobin binding (Fig. 1D and Table S5).

### KEGG pathway enrichment analysis for DEGs

**Figure 2:**
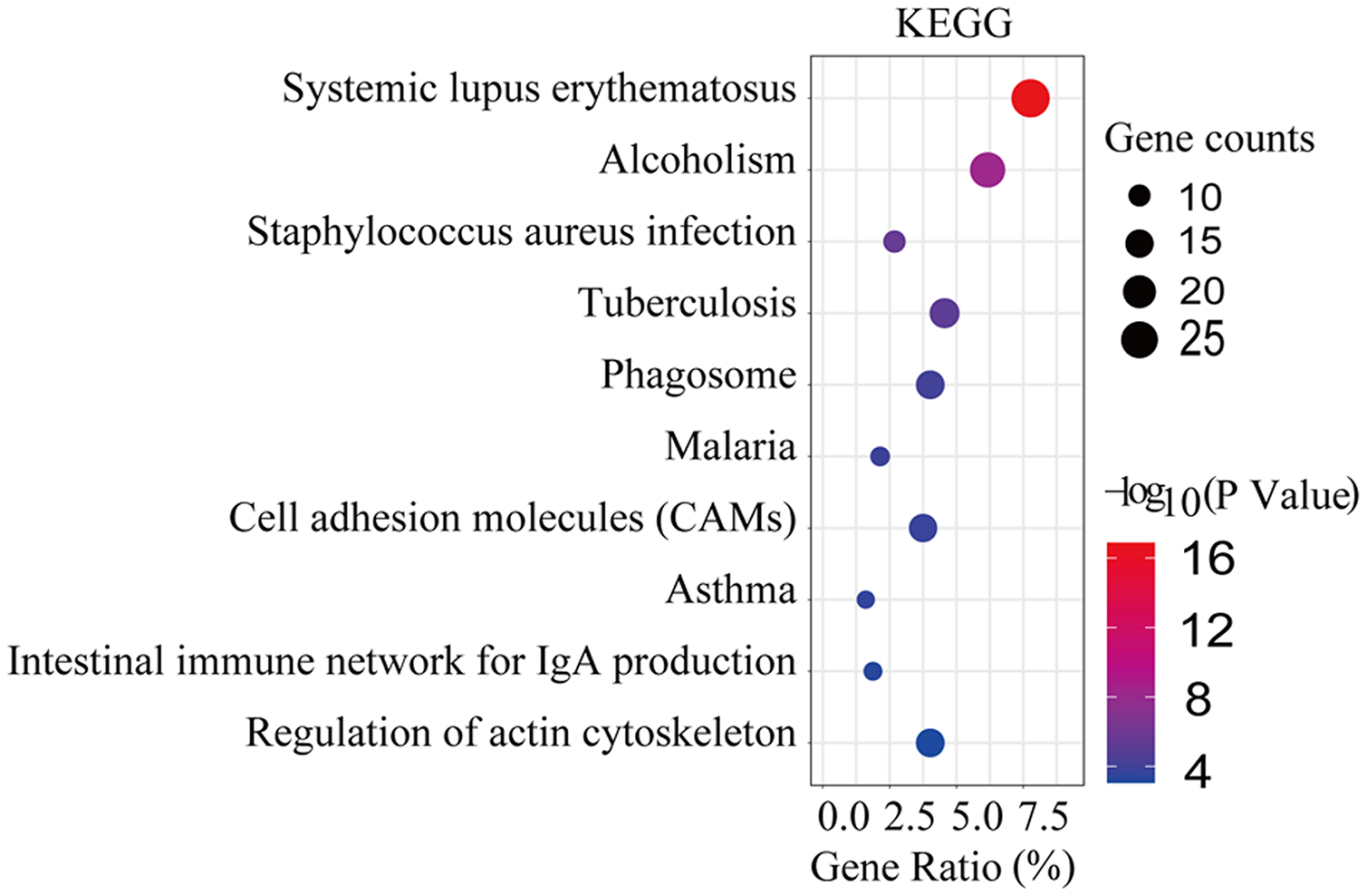
KEGG significant enrichment analysis of DEGs. The enrichment factor reflects the degree of enrichment of DGE in a given pathway. The number of enriched DGEs in the path is represented by the circled area, and the color of the circle represents the range of the corrected *P* value. Only the top 10 DEGs are shown here. Gene ratio: The gene ratio was defined as the ratio of the number of differential genes annotated on the KEGG pathway to the total number of differential genes.

KEGG pathway enrichment analysis was performed using DAVID (version 6.8). We observed that the DEGs were significantly enriched in systemic lupus erythematosus, alcoholism, staphylococcus aureus infection, tuberculosis, phagosome, malaria, cell adhesion molecules (CAMs), asthma, intestinal immune network for IgA production, and regulation of actin cytoskeleton (Fig. 2 and Table S6).

### PPI networks and prediction of TFs for DEGs in mouse

**Figure 3:**
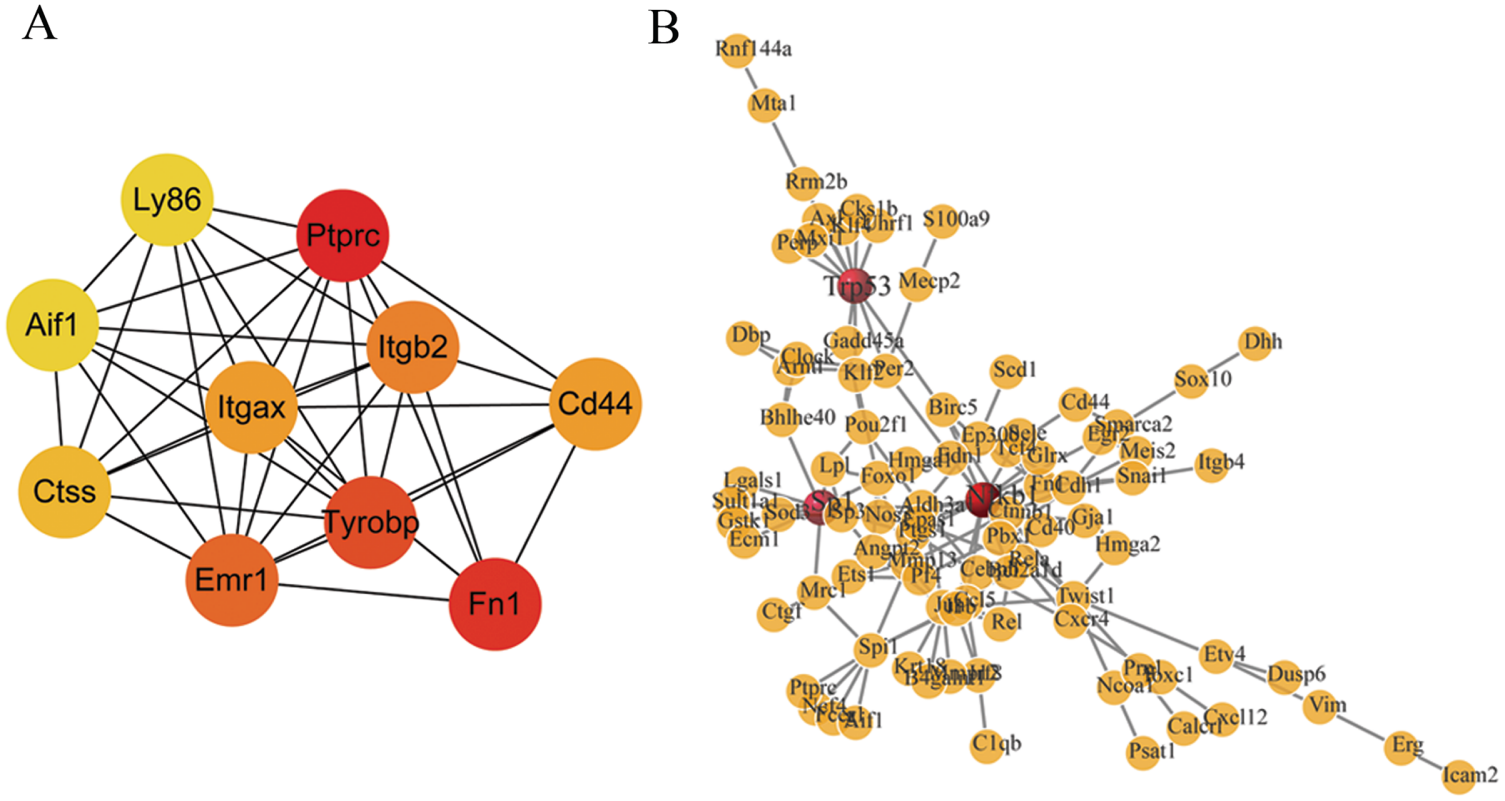
Major PPI networks and predicted transcription factors (TFs) of DEGs. (A) The major protein-protein interaction (PPI) network analysis of DEG. The color gradation represents the degree value; red represents a higher degree. (B) The prediction of mouse DEGs TFs. The red characters indicate that it is mostly TF. DEG, differentially expressed genes; TF, transcription factor.

We used STRING to construct an initial PPI network from 373 DEGs to study the interaction of these DEGs in the LCA/RCA gene set (Fig. S1). The initial PPI network was then imported into Cytoscape to construct a sub-network (Fig. 3A). A total of 10 candidate genes were identified as key genes and their edge degrees were more than 48 in the PPI analysis. According to the edge degree rank, the 10 key genes were *Ptprc* (degree = 73), *Fn1* (degree = 69), *Tyrobp* (degree = 59), *Emr1* (degree = 58), *Itgb2* (degree = 57), *Itgax* (degree = 54), *CD44* (degree = 54), *Ctss* (degree = 49), *Ly86* (degree = 48) and *Aif1* (degree = 48). TRRUST2.0 was used to analyze the promoter binding motifs and to identify the transcription factors associated with each gene. The results showed that Nfkb1, Sp1, and Trp53 transcription factor families played an important role in the regulation of the DEGs expression (Fig. 3B).

### Key gene validation

**Figure 4:**
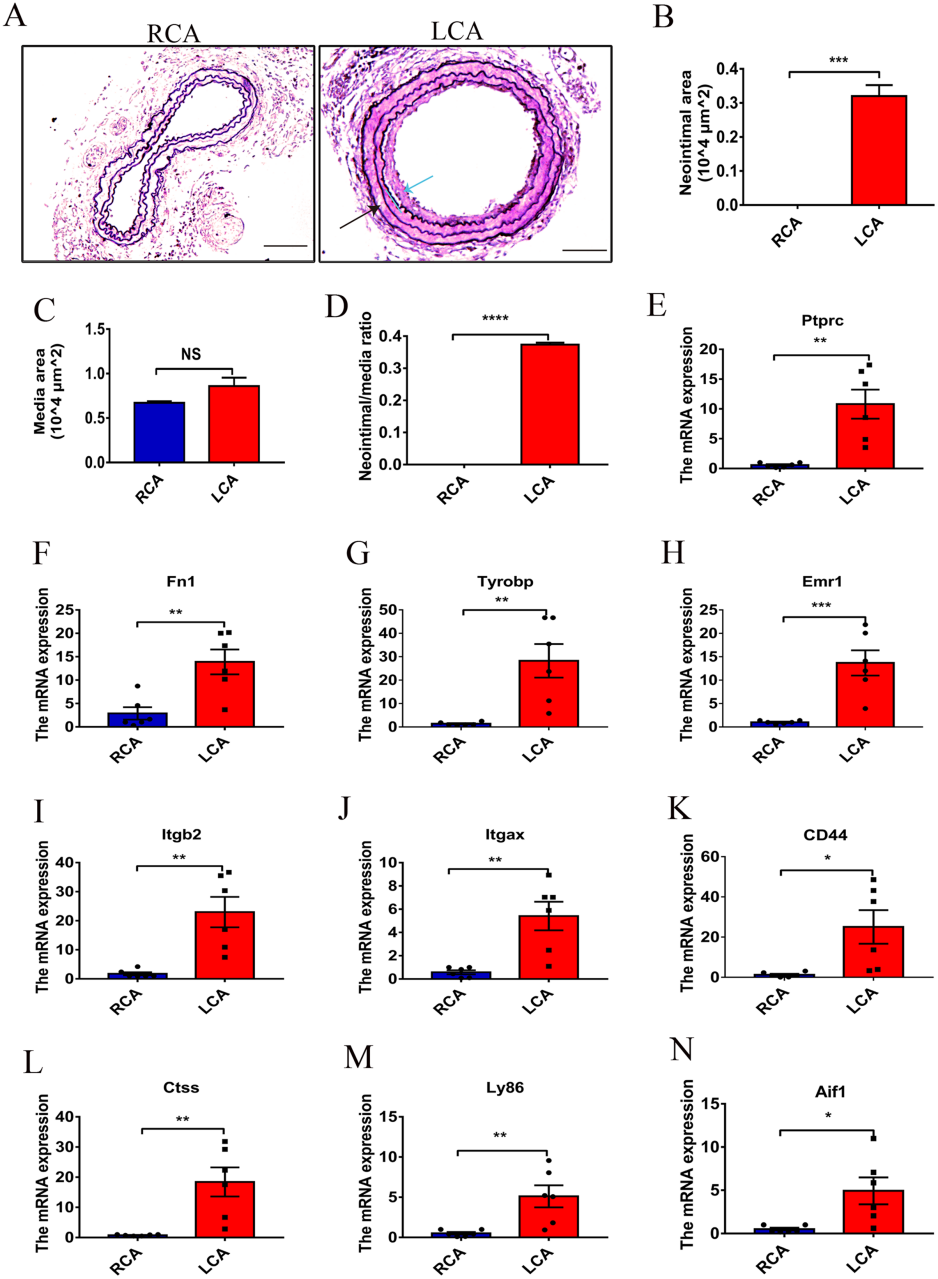
Verification of the top 10 key DEGs at the mRNA level. (A) Representative micrographs with Weigert staining of arterial sections from uninjured RCA (that underwent a sham operation) and injured LCA at 7 days after ligation. The black arrow represents the media of the carotid artery; the blue arrow represents the neointima of the carotid artery. Original magnification, 100×. Scale bar: 50 μm. (B) The quantitative analysis chart of neointimal area in RCA and LCA groups in mice. (C) The quantitative analysis chart of the media intima area in the RCA and the LCA groups in mice. (D) The quantitative analysis chart of neointima area/media area in RCA and LCA groups in mice. (A–D): Two-tailed unpaired Student’s *t*-test used to compare two groups. Data are expressed as means ± SEM. *n* = 3. *P < 0.05; **P < 0.01; ***P < 0.001; ****P < 0.0001. (E–N) The mRNA levels of Ptprc, Fn1, Tyrobp, Emr1, Itgb2, Itgax, CD44, Ctss, Ly86, and Aif1 in the carotid artery in the RCA and LCA groups in mice. All values have been standardized by GAPDH. Two-tailed unpaired Student’s *t*-test is used to compare two groups. Data are expressed as means ± SEM. *n* = 6. *P < 0.05; **P < 0.01; ***P < 0.001.

**Figure 5:**
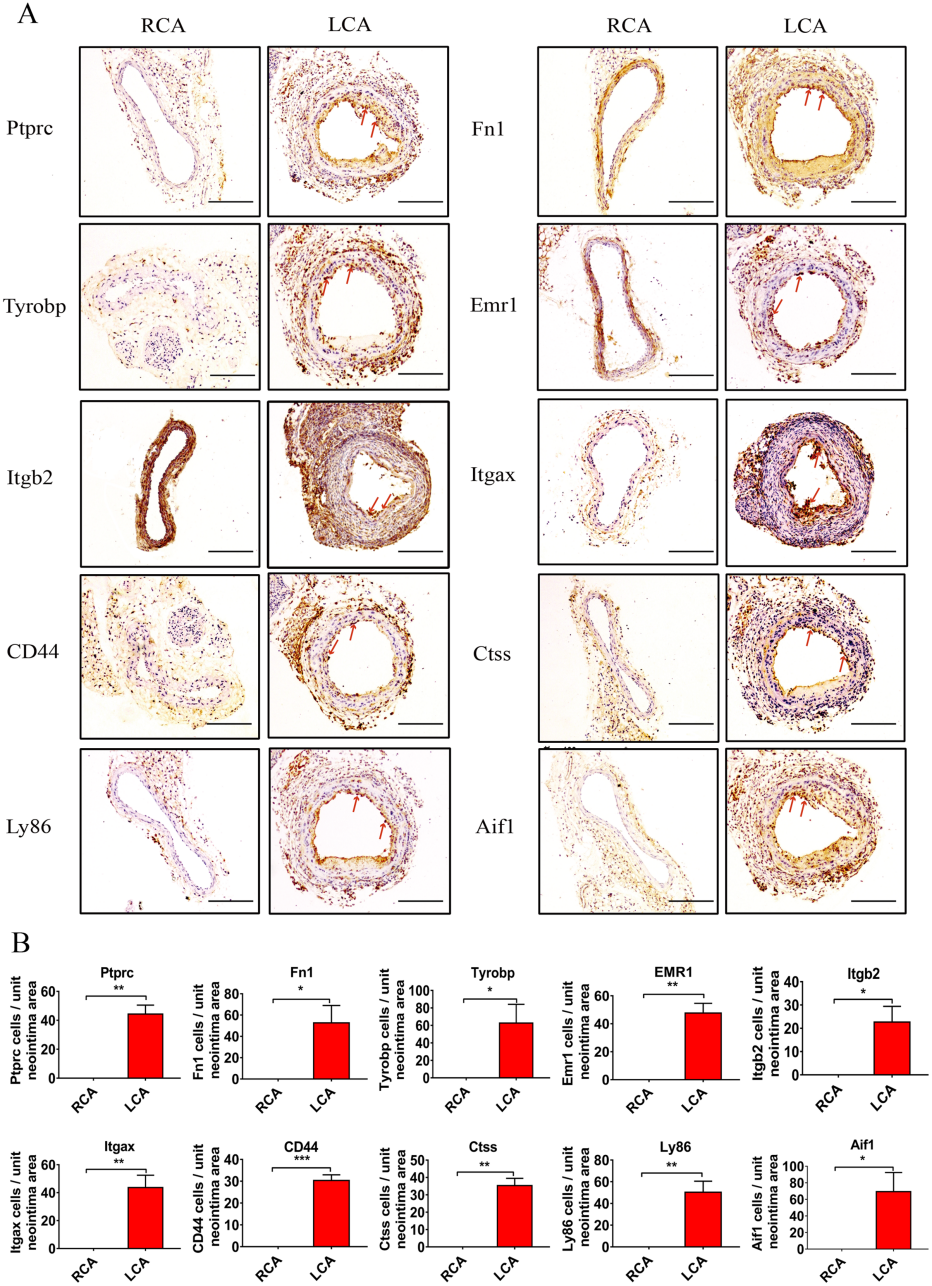
Verification of key DGEs at the protein level by immunohistochemistry. (A and B) Immunohistochemistry staining and quantitative analysis of Ptprc, Fn1, Tyrobp, Emr1, Itgb2, Itgax, CD44, Ctss, Ly86, and Aif1 proteins of carotid artery tissues. The arteries were harvested from uninjured RCA that underwent a sham operation, and injured LCA at 7 days after surgery. The red arrows represent positive cells. Two-tailed unpaired Student’s *t*-test is used to compare two groups. Data are expressed as means ± SEM. *n* = 3. *P < 0.05; **P < 0.01; ***P < 0.001; compared with the RCA group. Original magnification, 100×. Scale bar: 50 μm.

**Figure 6:**
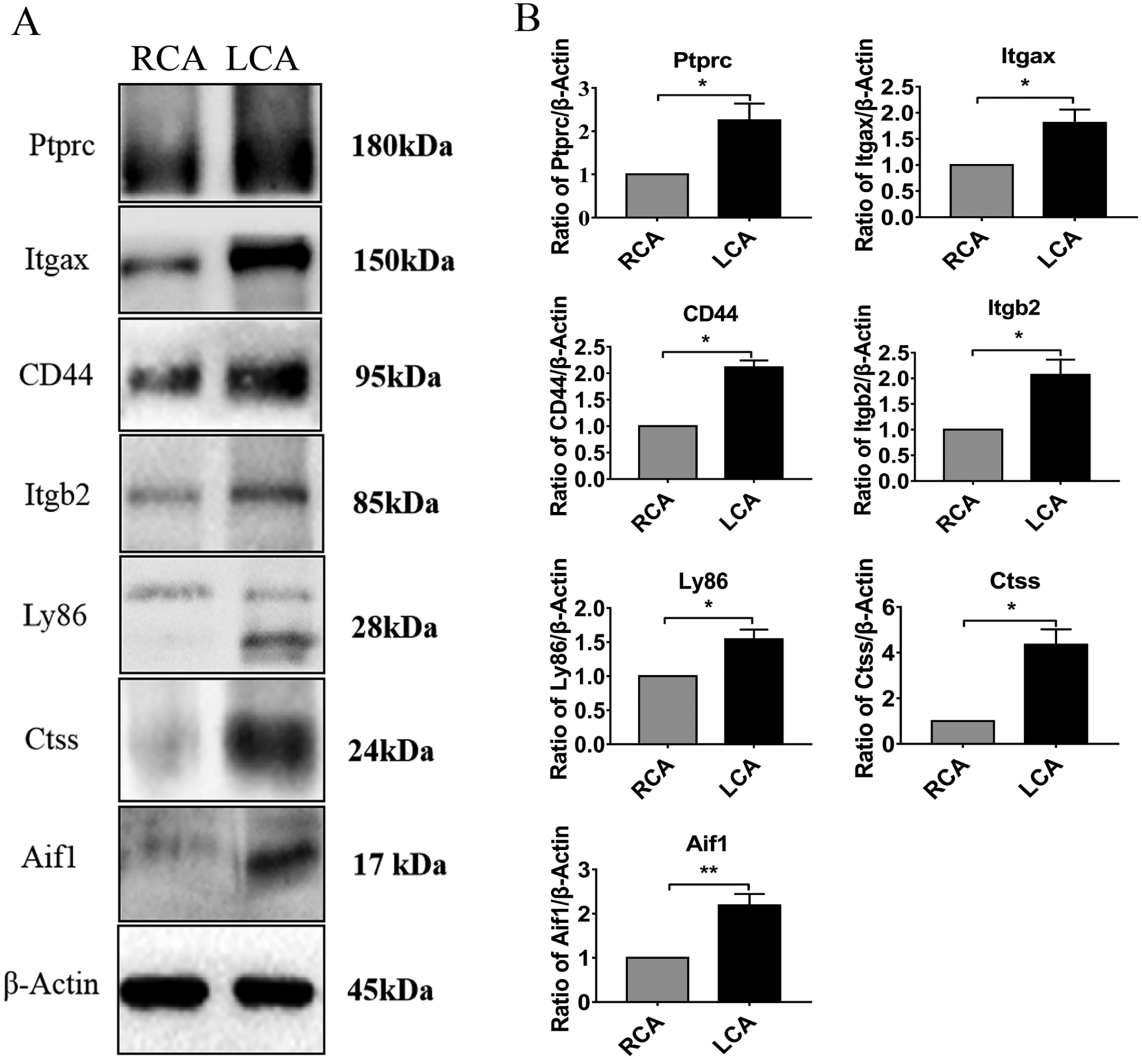
Verification of key DGEs at the protein level by western blot. (A and B) Representative western blotting and quantitative analysis of Ptprc, Itgax, CD44, Itgb2, Ly86, Ctss, and Aif1 proteins of carotid artery tissues. The arteries were harvested from uninjured RCA that underwent a sham operation, and injured LCA at 7 days after surgery. Two-tailed, unpaired Student’s *t*-test is used to compare two groups. Data are expressed as means ± SEM. *n* = 9. *P < 0.05; **P < 0.01; compared with the RCA group.

To further verify the results of the above bioinformatics analysis, we performed the carotid artery ligation in mice to establish a model of intimal hyperplasia. There was a certain degree of intimal formation in the carotid artery 7 days after ligation, as characterized by a slight increase in the thickness of the intima and the ratio of I/M compared with the RCA group (Figs. 4A–4D**)**. In addition, a small number of macrophages infiltrated in the carotid arteries 7 days after injury by immunohistochemistry (Fig. S2). RTqPCR analysis showed that the mRNA levels of Ptprc, Fn1, Tyrobp, Emr1, Itgb2, Itgax, CD44, Ctss, Ly86, and Aif1 in the intimal hyperplasia group were significantly increased (*P* < 0.05) compared to those in the RCA group. The results were consistent with the above-mentioned bioinformatic analysis (Figs. 4E–4N). In addition, the DEGs were further verified by immunohistochemistry (Fig. 5) and western blot analysis (Fig. 6), which revealed that the expression levels of these DEGs were upregulated in LCA mice compared with RCA mice 7 days after injury.

## Discussion

In this study, the global expression of DEGs was evaluated in intimal hyperplasia by analyzing the microarray dataset of RCA and LCA group in mice from the GEO database. A total of 373 DEGs were identified, including 199 upregulated genes and 174 downregulated genes. Furthermore, GO enrichment analysis and KEGG revealed the diversity of functions and pathways of DGEs. PPI network analysis showed that 10 DEGs with high node connectivity were selected as key genes. Three major transcription factors (*i.e*. Nfkb1, Sp1, Trp53) were predicted to potentially regulate the expression of the 373 DEGs. Finally, the expression changes of the key DEGs were verified at the mRNA and protein levels. Our bioinformatic analysis showed that the 10 key DEGs were closely related to immune inflammation and cell adhesion, despite not knowing the types of cells these DEGs originated from. Our previous studies demonstrated that that vascular smooth cells and infiltrated inflammatory cells play an essential role in the development of neointima formation after vascular injury (Du et al., 2013; Yao et al., 2018). Therefore, future studies will focus on determining the effects of key DEGs on certain types of cells and the development of vascular remodeling.

Accumulating evidence has shown that within hours to days following endothelial injury, inflammatory cells begin to infiltrate the injured area and secrete cytokines and growth factors. This has the potential to induce the migration and proliferation of VSMCs (Hansson, 2005). These cells secrete extracellular matrix components, including elastin and collagen, to form intimal hyperplasia and restenosis. Our KEGG pathway enrichment analysis indicated that cell adhesion molecules (CAMs) were enriched. Changes in the actin cytoskeleton are known as a prerequisite for vascular contractility and remodeling (Zhou, Gensch & Liao, 2011) and the regulation of the actin cytoskeleton is essential to maintaining vascular permeability, endothelial cell junction stability (Radeva & Waschke, 2018) and leukocyte migration (Marelli-Berg & Jangani, 2018). KEGG pathway analysis showed that the regulation of the actin cytoskeleton was significantly enriched.

However, after using PPI network analysis, only 10 up-regulated DEGs including Ptprc, Fn1, Tyrobp, Emr1, Itgb2, Itgax, CD44, Ctss, Ly86, and Aif1 were selected as key DEGs. RTqPCR, immunohistochemistry, and western blotting were used to detect the expression of these key DEGs at the mRNA and protein levels. We found that the mRNA and protein levels of key DEGs were also up-regulated in the carotid arteries of mice ligated for 7 days, which was consistent with the results of bioinformatics analysis.

Among the key DEGs, Ptprc (protein tyrosine phosphatase receptor type C), also known as CD45, can encode some members from the protein tyrosine phosphatase (PTP) family and plays a critical role in the process of cell growth, differentiation, mitosis, and carcinogenic transformation (Rheinlander, Schraven & Bommhardt, 2018). Previous studies have confirmed the role of CD45 on T cell receptor (TCR) and B cell receptor (BCR) signaling. The effects of CD45 in mast cells, macrophages, DCs and leucocyte adhesion and migration have been explored. The Janus kinases (JAKs) were identified as potential CD45 substrates (Saunders & Johnson, 2010). Fibronectin 1 (Fn1) encodes fibronectin as a glycoprotein on the cell surface and in the extracellular matrix, which is involved in cell adhesion and migration processes, including host defense, blood coagulation, metastasis and wound healing (Aota, Nomizu & Yamada, 1994; Aziz-Seible & Casey, 2011; Brentnall et al., 2014; Singh & Schwarzbauer, 2012; Veevers-Lowe et al., 2011). Fn1 is essential for smooth muscle cell phenotype regulation, proliferation, and cell adhesion (Tamura et al., 2000), and was implicated in vascular remodeling in radiation-induced brain injury (Andrews et al., 2017). Immune signaling adaptor TYROBP, also known as DAP12, encodes a transmembrane signaling polypeptide. This adaptor, initially characterized in NK cells, is associated with multiple cell-surface activating receptors expressed in both lymphoid and myeloid lineages and has multiple biological functions (Tomasello & Vivier, 2005). A previous study revealed that DAP12 signaling augmented the response to microbial products and amplified inflammation and thus contributed to mortality in sepsis (Turnbull et al., 2005). Consistently, a recent study of vascular transcriptomics demonstrated that the trend of TYROBP mRNA was increased in high fat/cholesterol (HFC) diet-fed Tibetan minipig atherosclerosis models (Pan et al., 2020).

Emr1, known as Adgre1, encodes the F4/80 antigen, which is restricted to leukocytes and plays an important role in the immune response by moderating the cell adhesion and inflammatory response. A previous study showed a trend of up-regulation for Emr1 in carotid artery balloon injury, consistent with our findings (Zhang et al., 2014). Additionally, the cytoplasmic region of Emr1 is required for regulating the number of ERMES foci, which regulates the number of foci of the endoplasmic reticulum-mitochondria encounter structure complex (Rasul et al., 2021). The integrin subunit beta2 (Itgb2), also known as CD18, is responsible for encoding an integrin beta chain and participating in cell adhesion and cell-surface mediated signaling. As a risk factor, Itgb2 had been shown to accelerate the development of myocardial infarction, atherothrombotic cerebral infarction (Lehmkuhl et al., 1996; May et al., 2002; Yamaguchi et al., 2006), and diabetic nephropathy (Geng et al., 2019) through the cell adhesion molecule pathway. Integrin subunit alpha X (Itgax), also known as CD11c, is a typical marker on the membrane of dendritic cells (DCs), which mediates cell-cell interaction during inflammatory responses. There is growing evidence that CD11c+DC may be involved in angiogenesis. Wu et al. (2009) showed that knocking out ITGAX in mice resulted in decreased vascular plaque formation, suggesting that ITGAX plays a pathogenic role of in atherogenesis. CD44, a cell adhesion molecule, is involved in angiogenesis, endothelial cell proliferation, and migration. It is a multi-faceted receptor, which exists in multiple activation states, variant isoforms, as well as intracellular and soluble forms (Pure & Cuff, 2001). Previous studies have shown that it plays an important role in the development of atherosclerotic lesions characterized by VSMC proliferation (Schultz, Rasmussen & Ledet, 2005; Zhao et al., 2008). In response to inflammation, CD44 is upregulated and functionally activated on vascular endothelial, smooth muscle and inflammatory cells (Johnson & Ruffell, 2009). We demonstrated that CD44 was significantly increased in the intimal hyperplasia from injured vessels. Notably, two previous studies provided strong evidence to support the protective role of CD44 in the pathological remodeling process (Vendrov et al., 2006; Zhao et al., 2011). Cysteine protease cathepsin S (Ctss) is mainly over-expressed in human and animal atherosclerosis and abdominal aortic aneurysms (Qin et al., 2013). Some studies have demonstrated that Ctss promotes neovascularization by producing pro-angiogenic factors, stimulates cell proliferation, and enhances the formation of endothelial capillary-like tubules (Premzl, Turk & Kos, 2006). Lymphocyte antigen 86 (Ly86), also known as MD1, has been shown to be involved in various pathophysiological processes including immune regulation, obesity, insulin resistance, and inflammation. Peng et al. (2017) demonstrated that the loss of MD1 aggravated the left ventricular structure and electrical remodeling in response to chronic pressure overload. Allogeneic transplantation inflammatory factor 1 (Aif1) encodes a protein that binds to actin and calcium, and may link inflammation with cell proliferation (Zhao, Yan & Chen, 2013). Several studies have shown that Aif1 and CD68+ macrophages co-localized in human atherosclerotic arteries (Tian, Kelemen & Autieri, 2006), and the severity of atherosclerosis was increased in Aif1 transgenic mice (Sommerville et al., 2012). These studies shown that these DEGs play an important role in immune inflammation, proliferation, migration, and adhesion of intimal hyperplasia.

We used the TRRUST2.0 to analyze the promoter binding motifs and to identify the transcription factors for the key genes in order to directly demonstrate the potential transcriptional regulation mechanism of the above-mentioned key genes in the intimal hyperplasia. We found that the most prominent transcription factor was Nfkb1, which regulates inflammatory response-related genes (Cartwright, Perkins & L. Wilson, 2016) and played a role in the neointima formation after vascular injury (Yoshimura et al., 2001). Additionally, Sp1 is involved in many cellular processes (O’Connor, Gilmour & Bonifer, 2016), including cell differentiation (Thomas et al., 2007), cell growth, apoptosis (Torabi et al., 2018), immune response (Kong et al., 2020), response to DNA damage (Bu et al., 2008), and chromatin remodeling (Cakouros et al., 2001). Sp1 plays an important role in the study of related mechanisms of intimal hyperplasia (Yang et al., 2013; Garrido-Martin et al., 2013). Trp53 responds to a variety of cellular stresses and is involved in the induction of cell cycle arrest, apoptosis, senescence (Levine & Oren, 2009; Freed-Pastor & Prives, 2012), and inhibited the intimal hyperplasia of the carotid artery in rats with balloon injury (Jacob, Hingorani & Ascher, 2012).

Interestingly, this dataset revealed that the deubiquitinating enzyme BRCC36 mRNA expression has an increased trend on the 7th day after surgery in the ligated arteries compared with the control arteries. By contrast, previous study showed that BRCC36 expression increased significantly in the 2th week (Fig. S3). The results suggested that the key DEGs may be involved in the dynamic regulation of intimal formation. Future studies should focus on addressing the detailed alternation, effects, and mechanism of the key DEGs in the development of vascular injury and remodeling. These studies may provide us some new insights in developing effective therapeutic strategies to prevent various vascular diseases such as coronary atherosclerosis, pulmonary hypertension, and restenosis. It is noted that the present study had some limitations. For example, the lack of more public data sets about intimal hyperplasia limited our detailed analysis, discussion, and verification. It is worth mentioning that the right carotid artery taken from an animal subjected to partial ligation of the left carotid artery was used as the control in the original study, and the original method-partial carotid ligation model in mice was adapted (Nam et al., 2009; Dunn et al., 2014). However, in our experiments, we conducted the complete ligation of the common carotid artery to explore a biological question not considered in the original publication, and the results are quite consistent. In addition, the right carotid artery taken from an animal subjected to ligation of the left carotid artery was also used as the control, which is a generally accepted method (Du et al., 2013; Yao et al., 2018; Godin et al., 2000).

## Conclusion

We revealed new insights into the underlying mechanism of vascular remodeling by exploring the key DEGs in the development of neointima formation. The results are promising and future study will focus on the key targets from the DEGs. A better understanding of their role will contribute to develop new therapeutic strategies in the prevention of vascular remodeling.

## Supplemental Information

10.7717/peerj.13436/supp-1 Supplemental Information 1 Supplementary tables.

10.7717/peerj.13436/supp-2 Supplemental Information 2 Author Checklist.

10.7717/peerj.13436/supp-3 Supplemental Information 3 rt-PCR raw data.

## Additional Information and Declarations

### Data Availability

The following information was supplied regarding data availability: The data is available in NCBI GEO: GES56143 and the original measured values are available in the Supplemental File.
