# Supplementary material for: Identification of key differential genes in intimal hyperplasia induced by left carotid artery ligation
Source: PeerJ. 2022 May 13;10:e13436. doi: 10.7717/peerj.13436 (PMC9109685; doi:10.7717/peerj.13436)
Supplement: Supplemental Information 1 [file peerj-10-13436-s001.docx]

**Supplementary materials 1**

**Table 1 Forward and reverse primer pairs.**

| **Gene names** | **Forward** | **Reverse** |
| --- | --- | --- |
| Ptprc  Fn1  Tyrobp  Emr1  Itgb2  Itgax  Cd44  CtSS  Ly86  Aif1 | GTGCCCATCAGTTCCTTACA  TCCTGTCTACCTCACAGACTAC  TGTGGTGTCCAGTGCATATC  ATTGCGGGATTCCTACACTATC  TTTACTTGCGACCAGGACAG  GTGCCCATCAGTTCCTTACA  GCAGAAATCAAGACGTTATGGG  GACGCTTCCTATCCCTACAAAG  TGGGTCACTCTTCCGATCTTA  GGAGTTTGATCTGAATGGAAATGG | GTCAGCTCCACAGTTCTTCTC  GTCTACTCCACCGAACAACAA  GACTTAATCCTCCCACAGTCAG  TTCACCACCTTCAGGTTTCTC  GAGGAGAGATCCATGAGGTAGT  GTCAGCTCCACAGTTCTTCTC  AAGCACCACCACCAAAGA  CCAAACGGGAGCTGAATGTA  ACTCAGGGTCCTCAGAAATAGA  TTCAGCTCTAGGTGGGTCTT |

**Table 2 Differentially expressed genes (DEGs).**

| ID | FC | pValue | adj.P.Val | t value | Gene Name |
| --- | --- | --- | --- | --- | --- |
| \| ILMN_1234487 \| \| --- \| \| ILMN_2638914 \| \| ILMN_2433119 \| \| ILMN_1230039 \| \| ILMN_3084087 \| \| ILMN_1239102 \| \| ILMN_2826916 \| \| ILMN_1217978 \| \| ILMN_1221817 \| \| ILMN_1225835 \| \| ILMN_1236393 \| \| ILMN_2518744 \| \| ILMN_2734729 \| \| ILMN_1240846 \| \| ILMN_2885990 \| \| ILMN_1238215 \| \| ILMN_3143483 \| \| ILMN_2451022 \| \| ILMN_1216953 \| \| ILMN_2631423 \| \| ILMN_1228314 \| \| ILMN_1249498 \| \| ILMN_1237224 \| \| ILMN_1250421 \| \| ILMN_2700848 \| \| ILMN_1243407 \| \| ILMN_2618408 \| \| ILMN_1218123 \| \| ILMN_1253370 \| \| ILMN_2854943 \| \| ILMN_2840430 \| \| ILMN_2742152 \| \| ILMN_1228031 \| \| ILMN_1253304 \| \| ILMN_2711267 \| \| ILMN_2737713 \| \| ILMN_2765224 \| \| ILMN_2728270 \| \| ILMN_2675760 \| \| ILMN_1222452 \| \| ILMN_2498731 \| \| ILMN_2750053 \| \| ILMN_1225699 \| \| ILMN_1253178 \| \| ILMN_2619983 \| \| ILMN_2595200 \| \| ILMN_2609998 \| \| ILMN_2653132 \| \| ILMN_3113303 \| \| ILMN_2931918 \| \| ILMN_2974798 \| \| ILMN_1242170 \| \| ILMN_2870522 \| \| ILMN_2970473 \| \| ILMN_1233424 \| \| ILMN_1244316 \| \| ILMN_1233127 \| \| ILMN_2643470 \| \| ILMN_1256369 \| \| ILMN_1235390 \| \| ILMN_2743013 \| \| ILMN_2681516 \| \| ILMN_2764580 \| \| ILMN_2472741 \| \| ILMN_1220170 \| \| ILMN_3008858 \| \| ILMN_1253813 \| \| ILMN_2651715 \| \| ILMN_2717439 \| \| ILMN_2999439 \| \| ILMN_2932964 \| \| ILMN_2693205 \| \| ILMN_2867147 \| \| ILMN_2677231 \| \| ILMN_1221199 \| \| ILMN_2715802 \| \| ILMN_2694955 \| \| ILMN_2772077 \| \| ILMN_2649333 \| \| ILMN_2596396 \| \| ILMN_2950422 \| \| ILMN_2629395 \| \| ILMN_1220234 \| \| ILMN_3060766 \| \| ILMN_1231814 \| \| ILMN_1246861 \| \| ILMN_2623983 \| \| ILMN_2980663 \| \| ILMN_1242427 \| \| ILMN_1236718 \| \| ILMN_1217849 \| \| ILMN_1247377 \| \| ILMN_1236188 \| \| ILMN_2829262 \| \| ILMN_2821916 \| \| ILMN_2700408 \| \| ILMN_2834379 \| \| ILMN_2596346 \| \| ILMN_2619620 \| \| ILMN_2588515 \| \| ILMN_2701321 \| \| ILMN_2631161 \| \| ILMN_1259418 \| \| ILMN_2971721 \| \| ILMN_2987862 \| \| ILMN_1249378 \| \| ILMN_2656422 \| \| ILMN_2942674 \| \| ILMN_2598103 \| \| ILMN_2664224 \| \| ILMN_3115796 \| \| ILMN_2592881 \| \| ILMN_1251390 \| \| ILMN_2725927 \| \| ILMN_2776431 \| \| ILMN_2618364 \| \| ILMN_2960308 \| \| ILMN_2707017 \| \| ILMN_2706730 \| \| ILMN_2590961 \| \| ILMN_2626359 \| \| ILMN_2668536 \| \| ILMN_2720836 \| \| ILMN_2714796 \| \| ILMN_2676022 \| \| ILMN_2993221 \| \| ILMN_1248367 \| \| ILMN_1213449 \| \| ILMN_2608133 \| \| ILMN_2642681 \| \| ILMN_2866856 \| \| ILMN_2660851 \| \| ILMN_2748875 \| \| ILMN_2856588 \| \| ILMN_2660099 \| \| ILMN_1230287 \| \| ILMN_1237375 \| \| ILMN_1229573 \| \| ILMN_2776922 \| \| ILMN_2790188 \| \| ILMN_1254692 \| \| ILMN_2622500 \| \| ILMN_1252481 \| \| ILMN_2671923 \| \| ILMN_1229155 \| \| ILMN_3112334 \| \| ILMN_1249486 \| \| ILMN_2459899 \| \| ILMN_2424060 \| \| ILMN_2715840 \| \| ILMN_1219017 \| \| ILMN_2745876 \| \| ILMN_2660555 \| \| ILMN_2604029 \| \| ILMN_1246486 \| \| ILMN_2628629 \| \| ILMN_2874853 \| \| ILMN_1254256 \| \| ILMN_2697433 \| \| ILMN_1254927 \| \| ILMN_2788593 \| \| ILMN_1220939 \| \| ILMN_2473620 \| \| ILMN_2767615 \| \| ILMN_1247947 \| \| ILMN_2994744 \| \| ILMN_2710159 \| \| ILMN_1239239 \| \| ILMN_2665535 \| \| ILMN_2663211 \| \| ILMN_2699488 \| \| ILMN_2513570 \| \| ILMN_1217021 \| \| ILMN_1254577 \| \| ILMN_1248139 \| \| ILMN_2940642 \| \| ILMN_2718662 \| \| ILMN_1232716 \| \| ILMN_2776008 \| \| ILMN_2768972 \| \| ILMN_2878071 \| \| ILMN_2510694 \| \| ILMN_2616226 \| \| ILMN_1230890 \| \| ILMN_1213850 \| \| ILMN_2657478 \| \| ILMN_2603725 \| \| ILMN_1238644 \| \| ILMN_2653385 \| \| ILMN_1228320 \| \| ILMN_2733185 \| \| ILMN_2671572 \| \| ILMN_2756704 \| \| ILMN_1239729 \| \| ILMN_1218949 \| \| ILMN_2934120 \| \| ILMN_2866185 \| \| ILMN_2772595 \| \| ILMN_2710274 \| \| ILMN_3145415 \| \| ILMN_1222602 \| \| ILMN_1216880 \| \| ILMN_2488360 \| \| ILMN_2745425 \| \| ILMN_2588682 \| \| ILMN_2433213 \| \| ILMN_2485526 \| \| ILMN_2508679 \| \| ILMN_2594031 \| \| ILMN_2915232 \| \| ILMN_2437254 \| \| ILMN_2803674 \| \| ILMN_3158250 \| \| ILMN_1260456 \| \| ILMN_2955919 \| \| ILMN_2500533 \| \| ILMN_2632712 \| \| ILMN_1255834 \| \| ILMN_2513960 \| \| ILMN_1239430 \| \| ILMN_1226395 \| \| ILMN_3114585 \| \| ILMN_1251634 \| \| ILMN_2861787 \| \| ILMN_1221067 \| \| ILMN_1217389 \| \| ILMN_2609323 \| \| ILMN_2503553 \| \| ILMN_1224077 \| \| ILMN_1219946 \| \| ILMN_1238603 \| \| ILMN_2939681 \| \| ILMN_2671056 \| \| ILMN_2737685 \| \| ILMN_2678127 \| \| ILMN_2759309 \| \| ILMN_2780280 \| \| ILMN_2614966 \| \| ILMN_2675232 \| \| ILMN_1256142 \| \| ILMN_1248441 \| \| ILMN_1226619 \| \| ILMN_2597769 \| \| ILMN_2595664 \| \| ILMN_1256676 \| \| ILMN_2699531 \| \| ILMN_3052632 \| \| ILMN_3144575 \| \| ILMN_2710419 \| \| ILMN_2947526 \| \| ILMN_2716935 \| \| ILMN_2543393 \| \| ILMN_1254031 \| \| ILMN_1239386 \| \| ILMN_2910934 \| \| ILMN_2660263 \| \| ILMN_2686975 \| \| ILMN_2936118 \| \| ILMN_2592253 \| \| ILMN_2793817 \| \| ILMN_1255739 \| \| ILMN_2865335 \| \| ILMN_1259400 \| \| ILMN_2670713 \| \| ILMN_2617433 \| \| ILMN_2419146 \| \| ILMN_1223257 \| \| ILMN_2757125 \| \| ILMN_1227458 \| \| ILMN_1248994 \| \| ILMN_2716185 \| \| ILMN_2704619 \| \| ILMN_1237186 \| \| ILMN_3143604 \| \| ILMN_1249637 \| \| ILMN_2958464 \| \| ILMN_1221048 \| \| ILMN_2480682 \| \| ILMN_2718217 \| \| ILMN_2686327 \| \| ILMN_1212836 \| \| ILMN_2688894 \| \| ILMN_2621752 \| \| ILMN_2678580 \| \| ILMN_2810882 \| \| ILMN_2692723 \| \| ILMN_2634235 \| \| ILMN_2669062 \| \| ILMN_2744164 \| \| ILMN_1216871 \| \| ILMN_1212702 \| \| ILMN_2759159 \| \| ILMN_2762528 \| \| ILMN_1219978 \| \| ILMN_2774121 \| \| ILMN_1244291 \| \| ILMN_1227907 \| \| ILMN_1228654 \| \| ILMN_1242661 \| \| ILMN_2630459 \| \| ILMN_2951691 \| \| ILMN_2457571 \| \| ILMN_2719139 \| \| ILMN_2646618 \| \| ILMN_1235158 \| \| ILMN_2777498 \| \| ILMN_2814974 \| \| ILMN_2956092 \| \| ILMN_2617228 \| \| ILMN_3006219 \| \| ILMN_2727172 \| \| ILMN_2723108 \| \| ILMN_2665441 \| \| ILMN_3118707 \| \| ILMN_2517041 \| \| ILMN_2619107 \| \| ILMN_1237038 \| \| ILMN_2588244 \| \| ILMN_2825574 \| \| ILMN_3003864 \| \| ILMN_2729958 \| \| ILMN_2655024 \| \| ILMN_2785454 \| \| ILMN_2769490 \| \| ILMN_2709211 \| \| ILMN_2765741 \| \| ILMN_2723639 \| \| ILMN_1221146 \| \| ILMN_2501489 \| \| ILMN_2888834 \| \| ILMN_1230357 \| \| ILMN_1235619 \| \| ILMN_1240938 \| \| ILMN_2723305 \| \| ILMN_2599192 \| \| ILMN_2777034 \| \| ILMN_1223317 \| \| ILMN_1253659 \| \| ILMN_1231012 \| \| ILMN_2622374 \| \| ILMN_2621448 \| \| ILMN_1256989 \| \| ILMN_2693991 \| \| ILMN_2983525 \| \| ILMN_1254276 \| \| ILMN_1233989 \| \| ILMN_1230157 \| \| ILMN_3132898 \| \| ILMN_1248830 \| \| ILMN_1219574 \| \| ILMN_2698449 \| \| ILMN_3040069 \| \| ILMN_2525565 \| \| ILMN_1234223 \| \| ILMN_1238276 \| \| ILMN_2757966 \| \| ILMN_2836654 \| \| ILMN_1227951 \| \| ILMN_1230708 \| \| ILMN_2638017 \| \| ILMN_1255416 \| \| ILMN_1245246 \| \| ILMN_2529842 \| \| ILMN_2661971 \| \| ILMN_2887986 \| \| ILMN_2847144 \| \| ILMN_1256476 \| \| ILMN_2985128 \| \| ILMN_2632034 \| \| ILMN_1226839 \| \| ILMN_1241892 \| \| ILMN_2745370 \| \| ILMN_2731901 \| | \| 1.99 \| \| --- \| \| -2.57 \| \| -1 \| \| -1.23 \| \| 1.31 \| \| 1.88 \| \| 1.04 \| \| -1.01 \| \| 3.09 \| \| -1.59 \| \| 1.11 \| \| -1.25 \| \| 2.34 \| \| -1.5 \| \| -1.39 \| \| 1.88 \| \| 2.76 \| \| 1.07 \| \| -1.05 \| \| 2.82 \| \| -1.25 \| \| 1.57 \| \| -1.55 \| \| 2.07 \| \| 1.62 \| \| -2.47 \| \| -1.4 \| \| 3.5 \| \| 1.4 \| \| -1.23 \| \| -1.25 \| \| 1.1 \| \| -1.46 \| \| -1.35 \| \| 1.26 \| \| 1.01 \| \| -1.36 \| \| 1.33 \| \| -1.27 \| \| -1.06 \| \| -1.38 \| \| -1.25 \| \| -1.76 \| \| -2.36 \| \| -1.05 \| \| -1.16 \| \| -1.18 \| \| 2.31 \| \| -1.35 \| \| -1.05 \| \| 1.15 \| \| -1.69 \| \| -1.77 \| \| 1.08 \| \| -1 \| \| 2.58 \| \| -1.08 \| \| 1.39 \| \| -1.37 \| \| -1.13 \| \| 1.08 \| \| 1.56 \| \| 1.18 \| \| -1.33 \| \| -1.08 \| \| 1.88 \| \| 1.64 \| \| 1.36 \| \| 1.41 \| \| -1.31 \| \| 1 \| \| 1.2 \| \| 3.08 \| \| -1.17 \| \| -1.31 \| \| 1.05 \| \| 1.31 \| \| 1.12 \| \| -1.36 \| \| -1.33 \| \| -1.19 \| \| -1.25 \| \| -1.34 \| \| -1.42 \| \| 1.91 \| \| 2.39 \| \| 1.02 \| \| -1.31 \| \| -1.2 \| \| 2.04 \| \| 3.4 \| \| 2.41 \| \| -1.04 \| \| -1.11 \| \| -1.2 \| \| -1.2 \| \| 2.13 \| \| 1.6 \| \| 2.49 \| \| -1.04 \| \| 1.2 \| \| 1.79 \| \| -1.04 \| \| -1.04 \| \| -1.02 \| \| -1.2 \| \| 1.68 \| \| -1.83 \| \| -1.6 \| \| -1.41 \| \| -1.09 \| \| -1.89 \| \| 1.55 \| \| 1.54 \| \| 1.56 \| \| 1.25 \| \| 1.05 \| \| -1.1 \| \| -1.35 \| \| -1.01 \| \| -1.11 \| \| -1.02 \| \| 1.43 \| \| 2.13 \| \| -1.03 \| \| -1.12 \| \| -1.56 \| \| -1.08 \| \| -2.03 \| \| 1.08 \| \| 1.22 \| \| 1.08 \| \| 2.09 \| \| -1.21 \| \| 1.03 \| \| 1.25 \| \| -1.23 \| \| 1.59 \| \| 1.21 \| \| -1.78 \| \| 1.79 \| \| -1.08 \| \| 1.25 \| \| 1.12 \| \| 1.04 \| \| -1.36 \| \| 2.8 \| \| -1.41 \| \| -1.56 \| \| 1.92 \| \| -1 \| \| -1.26 \| \| 2.26 \| \| -1.06 \| \| -1.32 \| \| 1.1 \| \| 1.11 \| \| -1.3 \| \| -1.05 \| \| -1.7 \| \| -1.34 \| \| -1.02 \| \| -1.1 \| \| -1.71 \| \| -1.32 \| \| -1.26 \| \| -1.03 \| \| -1.3 \| \| -1.79 \| \| -1.04 \| \| -1.54 \| \| -1.26 \| \| -1.04 \| \| 1.4 \| \| 1.26 \| \| -1.82 \| \| 1.17 \| \| -1.68 \| \| -1.26 \| \| -1.67 \| \| 2.86 \| \| -2.17 \| \| -1.62 \| \| -1.19 \| \| -1.98 \| \| 1.87 \| \| 1.18 \| \| -1.02 \| \| 1.18 \| \| 1.73 \| \| -1.27 \| \| 1.04 \| \| -1.37 \| \| -1.33 \| \| -1.54 \| \| 1.86 \| \| 1.09 \| \| 1.05 \| \| -1.47 \| \| 1.01 \| \| 1.21 \| \| 2.09 \| \| -1.42 \| \| 1.28 \| \| -1.68 \| \| -1.04 \| \| -1.36 \| \| 1.13 \| \| -1.01 \| \| 1.66 \| \| -1.02 \| \| 2.14 \| \| 1.1 \| \| -1.12 \| \| 1.25 \| \| -1.36 \| \| 1.19 \| \| 1.05 \| \| -1.59 \| \| 1.19 \| \| -1.46 \| \| 1.02 \| \| -1.81 \| \| 1.73 \| \| 1.08 \| \| 1.21 \| \| 1.22 \| \| 1.87 \| \| 1.29 \| \| 1.24 \| \| -1.49 \| \| 2.61 \| \| 1.35 \| \| 2.51 \| \| -1.04 \| \| 1.03 \| \| -1.06 \| \| -1.18 \| \| -2.08 \| \| 1.86 \| \| -1.24 \| \| 1.06 \| \| -1.37 \| \| -1.49 \| \| 1.07 \| \| 1.84 \| \| -1.05 \| \| -1.04 \| \| -1.31 \| \| -1.21 \| \| 1.64 \| \| 1.39 \| \| -1.1 \| \| 1.17 \| \| 1.71 \| \| 1.58 \| \| -1.02 \| \| 1.14 \| \| 1.97 \| \| -1.22 \| \| 1.49 \| \| -1.62 \| \| 1.07 \| \| 1.28 \| \| 1.17 \| \| 1.06 \| \| 1.15 \| \| 1.05 \| \| -1.17 \| \| -2.01 \| \| -1.7 \| \| -1.14 \| \| 1.64 \| \| 1.1 \| \| -1.19 \| \| 1.36 \| \| 1.07 \| \| -1.22 \| \| 1.28 \| \| -1.01 \| \| 1.62 \| \| 1.2 \| \| 1.33 \| \| 1.04 \| \| 1.16 \| \| 1.64 \| \| 1.28 \| \| -1.76 \| \| -1.19 \| \| 1.14 \| \| 1.45 \| \| 1.42 \| \| -1.09 \| \| -1.07 \| \| -1.14 \| \| 2.3 \| \| 1.14 \| \| 1.03 \| \| 1.41 \| \| 1.42 \| \| 2.34 \| \| -1.01 \| \| 1.1 \| \| 1.66 \| \| -1.17 \| \| 1.62 \| \| 1.75 \| \| 1.04 \| \| 1.1 \| \| 1.33 \| \| 1.2 \| \| -1.36 \| \| -1.76 \| \| 1.03 \| \| 1.37 \| \| 1.15 \| \| 1.46 \| \| 1.27 \| \| 1.26 \| \| -1.42 \| \| 2.1 \| \| 1.58 \| \| 1.08 \| \| 1.07 \| \| 1.24 \| \| -1.22 \| \| -1.1 \| \| -2.35 \| \| 1.17 \| \| 1.35 \| \| -1.6 \| \| -1.01 \| \| -1.21 \| \| 1.13 \| \| 1.02 \| \| 1.4 \| \| 1.84 \| \| 1.39 \| \| 1.26 \| \| -1.21 \| \| -1.03 \| \| 1.24 \| \| 1.05 \| \| 1.05 \| \| 1.17 \| \| 1.13 \| \| 1.12 \| \| -1.05 \| \| 1.71 \| \| 1.75 \| \| 1.32 \| \| 1.63 \| \| 1.51 \| \| 1.21 \| \| 1.64 \| \| 1.69 \| \| 1.95 \| \| 1.66 \| \| 1.6 \| \| -1.06 \| \| -1.69 \| \| -1.01 \| \| -1 \| \| 1.08 \| \| 1.24 \| \| 1.73 \| \| -1.29 \| \| 1.38 \| \| 1.59 \| \| 1.89 \| \| -1.26 \| \| -1.21 \| \| 1.14 \| | \| 1.19E-07 \| \| --- \| \| 3.08E-07 \| \| 6.31E-06 \| \| 1.21E-05 \| \| 2.51E-05 \| \| 2.96E-05 \| \| 4.49E-05 \| \| 4.72E-05 \| \| 4.97E-05 \| \| 6.29E-05 \| \| 7.08E-05 \| \| 1.02E-04 \| \| 1.11E-04 \| \| 1.13E-04 \| \| 1.22E-04 \| \| 1.22E-04 \| \| 1.24E-04 \| \| 1.33E-04 \| \| 1.34E-04 \| \| 1.38E-04 \| \| 1.48E-04 \| \| 1.98E-04 \| \| 1.98E-04 \| \| 2.07E-04 \| \| 2.38E-04 \| \| 2.42E-04 \| \| 2.49E-04 \| \| 2.64E-04 \| \| 2.72E-04 \| \| 3.07E-04 \| \| 3.12E-04 \| \| 3.42E-04 \| \| 3.65E-04 \| \| 4.09E-04 \| \| 4.61E-04 \| \| 4.68E-04 \| \| 4.75E-04 \| \| 4.95E-04 \| \| 5.69E-04 \| \| 5.75E-04 \| \| 6.04E-04 \| \| 6.14E-04 \| \| 6.20E-04 \| \| 6.93E-04 \| \| 7.13E-04 \| \| 7.29E-04 \| \| 7.40E-04 \| \| 7.42E-04 \| \| 7.66E-04 \| \| 7.81E-04 \| \| 8.00E-04 \| \| 8.21E-04 \| \| 9.32E-04 \| \| 9.33E-04 \| \| 9.37E-04 \| \| 1.00E-03 \| \| 1.01E-03 \| \| 1.05E-03 \| \| 1.13E-03 \| \| 1.13E-03 \| \| 1.15E-03 \| \| 1.16E-03 \| \| 1.17E-03 \| \| 1.17E-03 \| \| 1.23E-03 \| \| 1.28E-03 \| \| 1.28E-03 \| \| 1.33E-03 \| \| 1.33E-03 \| \| 1.37E-03 \| \| 1.44E-03 \| \| 1.50E-03 \| \| 1.52E-03 \| \| 1.55E-03 \| \| 1.56E-03 \| \| 1.57E-03 \| \| 1.69E-03 \| \| 1.69E-03 \| \| 1.71E-03 \| \| 1.72E-03 \| \| 1.76E-03 \| \| 1.78E-03 \| \| 1.85E-03 \| \| 1.89E-03 \| \| 1.90E-03 \| \| 1.96E-03 \| \| 2.04E-03 \| \| 2.07E-03 \| \| 2.11E-03 \| \| 2.31E-03 \| \| 2.34E-03 \| \| 2.38E-03 \| \| 2.57E-03 \| \| 2.67E-03 \| \| 2.75E-03 \| \| 2.78E-03 \| \| 2.78E-03 \| \| 2.86E-03 \| \| 3.05E-03 \| \| 3.05E-03 \| \| 3.09E-03 \| \| 3.27E-03 \| \| 3.31E-03 \| \| 3.44E-03 \| \| 3.46E-03 \| \| 3.49E-03 \| \| 3.50E-03 \| \| 3.62E-03 \| \| 3.71E-03 \| \| 3.82E-03 \| \| 3.88E-03 \| \| 4.06E-03 \| \| 4.10E-03 \| \| 4.18E-03 \| \| 4.21E-03 \| \| 4.22E-03 \| \| 4.35E-03 \| \| 4.36E-03 \| \| 4.61E-03 \| \| 4.66E-03 \| \| 4.74E-03 \| \| 4.77E-03 \| \| 4.89E-03 \| \| 5.10E-03 \| \| 5.18E-03 \| \| 5.20E-03 \| \| 5.23E-03 \| \| 5.38E-03 \| \| 5.55E-03 \| \| 5.65E-03 \| \| 5.93E-03 \| \| 6.03E-03 \| \| 6.09E-03 \| \| 6.14E-03 \| \| 6.18E-03 \| \| 6.18E-03 \| \| 6.52E-03 \| \| 6.62E-03 \| \| 6.64E-03 \| \| 6.69E-03 \| \| 6.90E-03 \| \| 6.99E-03 \| \| 7.06E-03 \| \| 7.25E-03 \| \| 7.38E-03 \| \| 7.51E-03 \| \| 7.54E-03 \| \| 7.56E-03 \| \| 7.58E-03 \| \| 7.67E-03 \| \| 7.74E-03 \| \| 7.88E-03 \| \| 7.94E-03 \| \| 7.96E-03 \| \| 7.97E-03 \| \| 8.03E-03 \| \| 8.06E-03 \| \| 8.85E-03 \| \| 9.25E-03 \| \| 9.34E-03 \| \| 9.40E-03 \| \| 9.50E-03 \| \| 9.53E-03 \| \| 9.66E-03 \| \| 9.69E-03 \| \| 9.81E-03 \| \| 9.85E-03 \| \| 9.86E-03 \| \| 9.90E-03 \| \| 9.92E-03 \| \| 9.93E-03 \| \| 1.00E-02 \| \| 1.00E-02 \| \| 1.01E-02 \| \| 1.02E-02 \| \| 1.05E-02 \| \| 1.05E-02 \| \| 1.06E-02 \| \| 1.06E-02 \| \| 1.07E-02 \| \| 1.08E-02 \| \| 1.09E-02 \| \| 1.12E-02 \| \| 1.13E-02 \| \| 1.15E-02 \| \| 1.15E-02 \| \| 1.17E-02 \| \| 1.18E-02 \| \| 1.19E-02 \| \| 1.19E-02 \| \| 1.20E-02 \| \| 1.23E-02 \| \| 1.23E-02 \| \| 1.23E-02 \| \| 1.24E-02 \| \| 1.24E-02 \| \| 1.24E-02 \| \| 1.25E-02 \| \| 1.25E-02 \| \| 1.26E-02 \| \| 1.27E-02 \| \| 1.27E-02 \| \| 1.29E-02 \| \| 1.30E-02 \| \| 1.30E-02 \| \| 1.30E-02 \| \| 1.31E-02 \| \| 1.31E-02 \| \| 1.32E-02 \| \| 1.34E-02 \| \| 1.35E-02 \| \| 1.37E-02 \| \| 1.40E-02 \| \| 1.41E-02 \| \| 1.44E-02 \| \| 1.46E-02 \| \| 1.46E-02 \| \| 1.47E-02 \| \| 1.47E-02 \| \| 1.47E-02 \| \| 1.49E-02 \| \| 1.51E-02 \| \| 1.52E-02 \| \| 1.52E-02 \| \| 1.54E-02 \| \| 1.55E-02 \| \| 1.57E-02 \| \| 1.58E-02 \| \| 1.60E-02 \| \| 1.61E-02 \| \| 1.61E-02 \| \| 1.62E-02 \| \| 1.63E-02 \| \| 1.64E-02 \| \| 1.65E-02 \| \| 1.67E-02 \| \| 1.67E-02 \| \| 1.68E-02 \| \| 1.69E-02 \| \| 1.72E-02 \| \| 1.73E-02 \| \| 1.73E-02 \| \| 1.73E-02 \| \| 1.78E-02 \| \| 1.78E-02 \| \| 1.80E-02 \| \| 1.81E-02 \| \| 1.81E-02 \| \| 1.81E-02 \| \| 1.82E-02 \| \| 1.83E-02 \| \| 1.84E-02 \| \| 1.86E-02 \| \| 1.87E-02 \| \| 1.88E-02 \| \| 1.88E-02 \| \| 1.89E-02 \| \| 1.93E-02 \| \| 1.93E-02 \| \| 1.94E-02 \| \| 1.99E-02 \| \| 2.01E-02 \| \| 2.01E-02 \| \| 2.02E-02 \| \| 2.02E-02 \| \| 2.03E-02 \| \| 2.05E-02 \| \| 2.06E-02 \| \| 2.09E-02 \| \| 2.14E-02 \| \| 2.16E-02 \| \| 2.19E-02 \| \| 2.22E-02 \| \| 2.24E-02 \| \| 2.29E-02 \| \| 2.32E-02 \| \| 2.33E-02 \| \| 2.35E-02 \| \| 2.36E-02 \| \| 2.38E-02 \| \| 2.39E-02 \| \| 2.42E-02 \| \| 2.46E-02 \| \| 2.46E-02 \| \| 2.49E-02 \| \| 2.54E-02 \| \| 2.54E-02 \| \| 2.55E-02 \| \| 2.56E-02 \| \| 2.58E-02 \| \| 2.61E-02 \| \| 2.61E-02 \| \| 2.63E-02 \| \| 2.63E-02 \| \| 2.65E-02 \| \| 2.76E-02 \| \| 2.76E-02 \| \| 2.77E-02 \| \| 2.79E-02 \| \| 2.86E-02 \| \| 2.90E-02 \| \| 2.90E-02 \| \| 2.91E-02 \| \| 2.96E-02 \| \| 2.97E-02 \| \| 3.01E-02 \| \| 3.01E-02 \| \| 3.14E-02 \| \| 3.14E-02 \| \| 3.19E-02 \| \| 3.23E-02 \| \| 3.24E-02 \| \| 3.25E-02 \| \| 3.29E-02 \| \| 3.30E-02 \| \| 3.32E-02 \| \| 3.33E-02 \| \| 3.33E-02 \| \| 3.34E-02 \| \| 3.38E-02 \| \| 3.39E-02 \| \| 3.41E-02 \| \| 3.45E-02 \| \| 3.45E-02 \| \| 3.46E-02 \| \| 3.57E-02 \| \| 3.58E-02 \| \| 3.59E-02 \| \| 3.70E-02 \| \| 3.71E-02 \| \| 3.72E-02 \| \| 3.72E-02 \| \| 3.73E-02 \| \| 3.73E-02 \| \| 3.80E-02 \| \| 3.82E-02 \| \| 3.86E-02 \| \| 3.87E-02 \| \| 3.95E-02 \| \| 3.96E-02 \| \| 3.97E-02 \| \| 4.00E-02 \| \| 4.02E-02 \| \| 4.03E-02 \| \| 4.08E-02 \| \| 4.13E-02 \| \| 4.15E-02 \| \| 4.21E-02 \| \| 4.25E-02 \| \| 4.36E-02 \| \| 4.39E-02 \| \| 4.42E-02 \| \| 4.43E-02 \| \| 4.46E-02 \| \| 4.54E-02 \| \| 4.56E-02 \| \| 4.57E-02 \| \| 4.64E-02 \| \| 4.68E-02 \| \| 4.75E-02 \| \| 4.79E-02 \| \| 4.81E-02 \| \| 4.82E-02 \| \| 4.83E-02 \| \| 4.84E-02 \| \| 4.84E-02 \| \| 4.86E-02 \| \| 4.86E-02 \| \| 4.89E-02 \| \| 4.93E-02 \| \| 4.96E-02 \| \| 4.98E-02 \| \| 4.98E-02 \| | \| 0.00367 \| \| --- \| \| 0.00465 \| \| 0.05223 \| \| 0.05223 \| \| 0.05296 \| \| 0.05328 \| \| 0.06092 \| \| 0.06092 \| \| 0.06092 \| \| 0.06407 \| \| 0.06407 \| \| 0.06463 \| \| 0.06463 \| \| 0.06463 \| \| 0.06555 \| \| 0.06555 \| \| 0.06555 \| \| 0.06555 \| \| 0.06555 \| \| 0.06555 \| \| 0.06607 \| \| 0.06758 \| \| 0.06758 \| \| 0.06994 \| \| 0.0719 \| \| 0.07205 \| \| 0.07332 \| \| 0.0746 \| \| 0.0746 \| \| 0.07752 \| \| 0.07752 \| \| 0.07966 \| \| 0.08032 \| \| 0.0831 \| \| 0.08491 \| \| 0.08515 \| \| 0.08555 \| \| 0.08555 \| \| 0.08912 \| \| 0.08912 \| \| 0.08912 \| \| 0.08912 \| \| 0.08939 \| \| 0.09225 \| \| 0.09363 \| \| 0.09412 \| \| 0.09462 \| \| 0.09462 \| \| 0.09486 \| \| 0.09511 \| \| 0.09588 \| \| 0.09652 \| \| 0.0992 \| \| 0.0992 \| \| 0.09921 \| \| 0.10152 \| \| 0.10152 \| \| 0.10205 \| \| 0.10555 \| \| 0.10563 \| \| 0.10679 \| \| 0.10704 \| \| 0.10704 \| \| 0.10704 \| \| 0.1078 \| \| 0.10802 \| \| 0.10802 \| \| 0.10874 \| \| 0.10874 \| \| 0.10908 \| \| 0.1116 \| \| 0.11287 \| \| 0.11306 \| \| 0.11315 \| \| 0.11315 \| \| 0.11345 \| \| 0.11753 \| \| 0.11753 \| \| 0.11759 \| \| 0.11759 \| \| 0.11759 \| \| 0.11774 \| \| 0.11806 \| \| 0.11806 \| \| 0.11806 \| \| 0.11885 \| \| 0.12078 \| \| 0.12162 \| \| 0.12318 \| \| 0.12687 \| \| 0.12712 \| \| 0.12812 \| \| 0.13294 \| \| 0.13545 \| \| 0.13675 \| \| 0.13693 \| \| 0.13693 \| \| 0.13777 \| \| 0.14142 \| \| 0.14142 \| \| 0.1423 \| \| 0.14653 \| \| 0.14702 \| \| 0.1503 \| \| 0.15083 \| \| 0.1515 \| \| 0.15168 \| \| 0.15325 \| \| 0.15468 \| \| 0.1568 \| \| 0.15719 \| \| 0.15959 \| \| 0.15979 \| \| 0.16022 \| \| 0.16022 \| \| 0.16023 \| \| 0.16168 \| \| 0.16168 \| \| 0.16581 \| \| 0.16609 \| \| 0.16738 \| \| 0.16752 \| \| 0.16881 \| \| 0.17071 \| \| 0.17124 \| \| 0.17131 \| \| 0.17165 \| \| 0.17351 \| \| 0.1736 \| \| 0.17555 \| \| 0.17727 \| \| 0.17812 \| \| 0.17874 \| \| 0.17929 \| \| 0.17959 \| \| 0.17959 \| \| 0.1834 \| \| 0.18454 \| \| 0.18476 \| \| 0.18515 \| \| 0.18646 \| \| 0.18731 \| \| 0.18828 \| \| 0.18992 \| \| 0.19158 \| \| 0.19222 \| \| 0.19222 \| \| 0.19222 \| \| 0.19223 \| \| 0.1932 \| \| 0.19395 \| \| 0.19555 \| \| 0.19632 \| \| 0.19681 \| \| 0.19682 \| \| 0.19748 \| \| 0.19748 \| \| 0.20544 \| \| 0.20907 \| \| 0.20992 \| \| 0.21071 \| \| 0.21163 \| \| 0.21185 \| \| 0.21235 \| \| 0.21253 \| \| 0.21322 \| \| 0.21331 \| \| 0.21331 \| \| 0.21331 \| \| 0.21331 \| \| 0.21334 \| \| 0.21425 \| \| 0.21425 \| \| 0.21468 \| \| 0.21546 \| \| 0.2175 \| \| 0.2178 \| \| 0.21839 \| \| 0.21839 \| \| 0.21927 \| \| 0.21953 \| \| 0.22012 \| \| 0.22152 \| \| 0.22263 \| \| 0.22349 \| \| 0.22349 \| \| 0.22525 \| \| 0.22525 \| \| 0.22603 \| \| 0.22603 \| \| 0.22612 \| \| 0.22775 \| \| 0.22777 \| \| 0.22777 \| \| 0.22779 \| \| 0.22779 \| \| 0.22779 \| \| 0.22779 \| \| 0.22781 \| \| 0.22837 \| \| 0.22904 \| \| 0.22904 \| \| 0.2306 \| \| 0.23085 \| \| 0.23085 \| \| 0.23085 \| \| 0.23181 \| \| 0.2323 \| \| 0.23254 \| \| 0.23395 \| \| 0.23448 \| \| 0.23547 \| \| 0.23682 \| \| 0.23754 \| \| 0.23883 \| \| 0.24006 \| \| 0.24012 \| \| 0.24056 \| \| 0.2407 \| \| 0.2407 \| \| 0.24172 \| \| 0.24247 \| \| 0.24255 \| \| 0.24263 \| \| 0.24278 \| \| 0.24367 \| \| 0.24409 \| \| 0.24592 \| \| 0.24667 \| \| 0.24755 \| \| 0.24755 \| \| 0.24782 \| \| 0.24785 \| \| 0.24785 \| \| 0.24904 \| \| 0.25016 \| \| 0.25049 \| \| 0.2507 \| \| 0.2507 \| \| 0.25186 \| \| 0.2521 \| \| 0.2521 \| \| 0.2521 \| \| 0.25398 \| \| 0.25407 \| \| 0.25537 \| \| 0.25568 \| \| 0.25568 \| \| 0.25569 \| \| 0.25631 \| \| 0.2565 \| \| 0.25658 \| \| 0.2583 \| \| 0.25869 \| \| 0.25887 \| \| 0.25887 \| \| 0.25937 \| \| 0.26145 \| \| 0.26145 \| \| 0.26174 \| \| 0.26507 \| \| 0.26546 \| \| 0.26546 \| \| 0.2659 \| \| 0.26618 \| \| 0.26635 \| \| 0.26759 \| \| 0.26759 \| \| 0.26845 \| \| 0.26987 \| \| 0.27082 \| \| 0.27189 \| \| 0.27329 \| \| 0.27413 \| \| 0.27669 \| \| 0.27845 \| \| 0.27845 \| \| 0.27915 \| \| 0.27964 \| \| 0.28092 \| \| 0.28115 \| \| 0.28259 \| \| 0.28437 \| \| 0.28477 \| \| 0.28546 \| \| 0.28689 \| \| 0.28689 \| \| 0.28733 \| \| 0.28733 \| \| 0.28895 \| \| 0.28992 \| \| 0.28992 \| \| 0.29087 \| \| 0.29087 \| \| 0.29174 \| \| 0.29641 \| \| 0.29683 \| \| 0.29695 \| \| 0.29747 \| \| 0.30064 \| \| 0.30232 \| \| 0.30256 \| \| 0.30259 \| \| 0.30521 \| \| 0.30521 \| \| 0.30751 \| \| 0.30751 \| \| 0.31236 \| \| 0.31241 \| \| 0.31421 \| \| 0.31544 \| \| 0.31577 \| \| 0.31591 \| \| 0.31733 \| \| 0.31774 \| \| 0.31812 \| \| 0.31836 \| \| 0.31841 \| \| 0.31865 \| \| 0.32037 \| \| 0.32062 \| \| 0.32117 \| \| 0.32243 \| \| 0.32284 \| \| 0.32284 \| \| 0.32574 \| \| 0.32574 \| \| 0.32592 \| \| 0.32901 \| \| 0.32901 \| \| 0.32929 \| \| 0.32935 \| \| 0.32946 \| \| 0.32946 \| \| 0.33162 \| \| 0.33237 \| \| 0.33308 \| \| 0.33312 \| \| 0.33614 \| \| 0.33624 \| \| 0.33671 \| \| 0.33762 \| \| 0.33807 \| \| 0.33856 \| \| 0.34016 \| \| 0.34122 \| \| 0.34177 \| \| 0.34393 \| \| 0.3447 \| \| 0.34767 \| \| 0.34825 \| \| 0.34895 \| \| 0.34923 \| \| 0.34998 \| \| 0.35213 \| \| 0.3526 \| \| 0.35281 \| \| 0.35467 \| \| 0.35558 \| \| 0.35784 \| \| 0.35866 \| \| 0.35936 \| \| 0.35971 \| \| 0.35984 \| \| 0.35988 \| \| 0.35988 \| \| 0.36032 \| \| 0.36032 \| \| 0.3611 \| \| 0.36202 \| \| 0.36244 \| \| 0.36277 \| \| 0.36295 \| | \| 3.55E+01 \| \| --- \| \| ###### \| \| ###### \| \| ###### \| \| 1.31E+1 \| \| 1.27E+1 \| \| 1.18E+01 \| \| ####### \| \| 1.15E+01 \| \| ####### \| \| 1.08E+01 \| \| ####### \| \| 9.89 \| \| -9.86 \| \| -9.71 \| \| 9.71 \| \| 9.68 \| \| 9.55 \| \| -9.54 \| \| 9.48 \| \| -9.36 \| \| 8.85 \| \| -8.84 \| \| 8.77 \| \| 8.53 \| \| -8.51 \| \| -8.46 \| \| 8.36 \| \| 8.31 \| \| -8.12 \| \| -8.09 \| \| 7.94 \| \| -7.84 \| \| -7.67 \| \| 7.49 \| \| 7.47 \| \| -7.44 \| \| 7.38 \| \| -7.18 \| \| -7.17 \| \| -7.1 \| \| -7.07 \| \| -7.06 \| \| -6.9 \| \| -6.86 \| \| -6.83 \| \| -6.81 \| \| 6.81 \| \| -6.76 \| \| -6.74 \| \| 6.7 \| \| -6.67 \| \| -6.5 \| \| 6.5 \| \| -6.49 \| \| 6.4 \| \| -6.4 \| \| 6.34 \| \| -6.25 \| \| -6.25 \| \| 6.23 \| \| 6.22 \| \| 6.2 \| \| -6.2 \| \| -6.14 \| \| 6.09 \| \| 6.08 \| \| 6.04 \| \| 6.04 \| \| -6 \| \| 5.94 \| \| 5.89 \| \| 5.88 \| \| -5.85 \| \| -5.84 \| \| 5.84 \| \| 5.75 \| \| 5.74 \| \| -5.73 \| \| -5.73 \| \| -5.7 \| \| -5.69 \| \| -5.64 \| \| -5.61 \| \| 5.61 \| \| 5.57 \| \| 5.53 \| \| -5.51 \| \| -5.48 \| \| 5.38 \| \| 5.36 \| \| 5.34 \| \| -5.26 \| \| -5.21 \| \| -5.18 \| \| -5.17 \| \| 5.17 \| \| 5.14 \| \| 5.07 \| \| -5.07 \| \| 5.05 \| \| 4.99 \| \| -4.98 \| \| -4.94 \| \| -4.93 \| \| -4.92 \| \| 4.92 \| \| -4.88 \| \| -4.86 \| \| -4.82 \| \| -4.81 \| \| -4.76 \| \| 4.75 \| \| 4.73 \| \| 4.72 \| \| 4.72 \| \| 4.69 \| \| -4.69 \| \| -4.63 \| \| -4.62 \| \| -4.6 \| \| -4.59 \| \| 4.57 \| \| 4.52 \| \| -4.51 \| \| -4.5 \| \| -4.5 \| \| -4.47 \| \| -4.44 \| \| 4.42 \| \| 4.37 \| \| 4.35 \| \| 4.35 \| \| -4.34 \| \| 4.33 \| \| 4.33 \| \| -4.28 \| \| 4.26 \| \| 4.26 \| \| -4.25 \| \| 4.22 \| \| -4.21 \| \| 4.2 \| \| 4.17 \| \| 4.16 \| \| -4.14 \| \| 4.14 \| \| -4.13 \| \| -4.13 \| \| 4.12 \| \| -4.11 \| \| -4.09 \| \| 4.09 \| \| -4.08 \| \| -4.08 \| \| 4.08 \| \| 4.07 \| \| -3.98 \| \| -3.94 \| \| -3.93 \| \| -3.93 \| \| -3.92 \| \| -3.91 \| \| -3.9 \| \| -3.9 \| \| -3.89 \| \| -3.88 \| \| -3.88 \| \| -3.88 \| \| -3.88 \| \| -3.88 \| \| -3.87 \| \| -3.87 \| \| 3.86 \| \| 3.85 \| \| -3.83 \| \| 3.82 \| \| -3.82 \| \| -3.82 \| \| -3.81 \| \| 3.8 \| \| -3.79 \| \| -3.77 \| \| -3.75 \| \| -3.74 \| \| 3.74 \| \| 3.72 \| \| -3.72 \| \| 3.71 \| \| 3.71 \| \| -3.7 \| \| 3.68 \| \| -3.68 \| \| -3.68 \| \| -3.67 \| \| 3.67 \| \| 3.67 \| \| 3.67 \| \| -3.66 \| \| 3.66 \| \| 3.65 \| \| 3.65 \| \| -3.64 \| \| 3.63 \| \| -3.63 \| \| -3.63 \| \| -3.63 \| \| 3.62 \| \| -3.62 \| \| 3.61 \| \| -3.6 \| \| 3.58 \| \| 3.57 \| \| -3.56 \| \| 3.54 \| \| -3.53 \| \| 3.53 \| \| 3.52 \| \| -3.52 \| \| 3.52 \| \| -3.51 \| \| 3.5 \| \| -3.49 \| \| 3.49 \| \| 3.48 \| \| 3.47 \| \| 3.47 \| \| 3.46 \| \| 3.45 \| \| 3.44 \| \| -3.44 \| \| 3.43 \| \| 3.43 \| \| 3.43 \| \| -3.42 \| \| 3.41 \| \| -3.41 \| \| -3.4 \| \| -3.4 \| \| 3.38 \| \| -3.38 \| \| 3.38 \| \| -3.38 \| \| -3.36 \| \| 3.35 \| \| 3.34 \| \| -3.34 \| \| -3.34 \| \| -3.34 \| \| -3.34 \| \| 3.33 \| \| 3.33 \| \| -3.31 \| \| 3.31 \| \| 3.31 \| \| 3.31 \| \| -3.3 \| \| 3.28 \| \| 3.28 \| \| -3.28 \| \| 3.26 \| \| -3.25 \| \| 3.25 \| \| 3.25 \| \| 3.24 \| \| 3.24 \| \| 3.23 \| \| 3.23 \| \| -3.22 \| \| -3.2 \| \| -3.19 \| \| -3.18 \| \| 3.16 \| \| 3.16 \| \| -3.14 \| \| 3.13 \| \| 3.13 \| \| -3.12 \| \| 3.11 \| \| -3.11 \| \| 3.1 \| \| 3.09 \| \| 3.08 \| \| 3.08 \| \| 3.07 \| \| 3.05 \| \| 3.05 \| \| -3.05 \| \| -3.05 \| \| 3.04 \| \| 3.03 \| \| 3.03 \| \| -3.03 \| \| -3.03 \| \| -3.02 \| \| 2.99 \| \| 2.98 \| \| 2.98 \| \| 2.98 \| \| 2.95 \| \| 2.95 \| \| -2.94 \| \| 2.94 \| \| 2.93 \| \| -2.93 \| \| 2.91 \| \| 2.91 \| \| 2.88 \| \| 2.88 \| \| 2.87 \| \| 2.86 \| \| -2.85 \| \| -2.85 \| \| 2.84 \| \| 2.84 \| \| 2.83 \| \| 2.83 \| \| 2.83 \| \| 2.83 \| \| -2.82 \| \| 2.82 \| \| 2.81 \| \| 2.8 \| \| 2.8 \| \| 2.8 \| \| -2.77 \| \| -2.77 \| \| -2.77 \| \| 2.75 \| \| 2.75 \| \| -2.74 \| \| -2.74 \| \| -2.74 \| \| 2.74 \| \| 2.73 \| \| 2.72 \| \| 2.71 \| \| 2.71 \| \| 2.69 \| \| -2.69 \| \| -2.69 \| \| 2.68 \| \| 2.68 \| \| 2.68 \| \| 2.67 \| \| 2.66 \| \| 2.66 \| \| -2.64 \| \| 2.64 \| \| 2.62 \| \| 2.61 \| \| 2.61 \| \| 2.6 \| \| 2.6 \| \| 2.58 \| \| 2.58 \| \| 2.58 \| \| 2.57 \| \| 2.56 \| \| -2.55 \| \| -2.54 \| \| -2.54 \| \| -2.54 \| \| 2.54 \| \| 2.53 \| \| 2.53 \| \| -2.53 \| \| 2.53 \| \| 2.53 \| \| 2.52 \| \| -2.51 \| \| -2.51 \| \| 2.51 \| | \| Angpt2 \| \| --- \| \| Prrt4 \| \| Syt7 \| \| Hyal2 \| \| Ivns1abp \| \| H2-Eb1 \| \| Ptpre \| \| Hoxa5 \| \| Cd74 \| \| Mfap5 \| \| Cd180 \| \| Wnk4 \| \| H2-Aa \| \| Fndc1 \| \| Pdlim3 \| \| Ctgf \| \| Lat2 \| \| Vim \| \| Prmt2 \| \| H2-Ab1 \| \| Col4a4 \| \| Plek \| \| Kctd12 \| \| Mmp12 \| \| Arrb2 \| \| Klk10 \| \| Icam2 \| \| Aif1 \| \| Arhgap20 \| \| Gprc5a \| \| Fam20a \| \| Gadd45a \| \| Dusp8 \| \| Stmn2 \| \| Krt18 \| \| Edn1 \| \| Bcam \| \| B4galnt1 \| \| Slc52a3 \| \| Itpr2 \| \| Eln \| \| Ptprj \| \| Serpina1a \| \| Aldh3a1 \| \| Sdc2 \| \| Cxx1a \| \| Fxyd6 \| \| Clec7a \| \| Atp2b2 \| \| Nxpe2 \| \| Lamc1 \| \| Adra2a \| \| Plekha6 \| \| Npm1 \| \| Arhgef2 \| \| Hbb-bs \| \| Rfwd2 \| \| Depdc7 \| \| Lynx1 \| \| Calcrl \| \| Ncf4 \| \| Slc39a6 \| \| Parvg \| \| Tpbgl \| \| Tek \| \| Ctsc \| \| Nckap1l \| \| Axl \| \| Pip4k2a \| \| Klf4 \| \| Ctps \| \| Aspm \| \| Tyrobp \| \| Hist1h2bc \| \| Ppm1f \| \| Hmga1 \| \| Igfbp4 \| \| Bok \| \| Rps6kl1 \| \| Grasp \| \| Tppp \| \| Rhoq \| \| Serpina1e \| \| Atl2 \| \| Ccl5 \| \| Ctss \| \| Egr2 \| \| Aqp1 \| \| Itga9 \| \| Hbb-bt \| \| Laptm5 \| \| Mpeg1 \| \| Twist1 \| \| Ltbp4 \| \| Nicn1 \| \| Mgll \| \| Tgfbi \| \| Dcn \| \| C1qb \| \| Phf1 \| \| Dusp6 \| \| Fcgr4 \| \| Aifm2 \| \| Fbrsl1 \| \| Per2 \| \| Bhlhe40 \| \| BC028528 \| \| Lims2 \| \| Emp2 \| \| Ephx1 \| \| Cd40 \| \| Jam2 \| \| Tbxas1 \| \| Serpina3g \| \| C1qa \| \| Flnc \| \| Hspe1 \| \| Sec14l2 \| \| Ptprr \| \| Hadh \| \| Cds2 \| \| Efcab14 \| \| Rbm47 \| \| Coro1a \| \| Kitl \| \| Slc4a4 \| \| Abat \| \| Man2c1 \| \| Rhpn2 \| \| Atf4 \| \| H2-DMa \| \| Bcl10 \| \| Fcer1g \| \| Gkn3 \| \| Slc25a5 \| \| Cd300ld \| \| Scd1 \| \| Neurl3 \| \| Glrx \| \| Fam135a \| \| Ms4a6b \| \| Zbtb7c \| \| Fosl2 \| \| Ly86 \| \| Sele \| \| Pitpnm3 \| \| Clec10a \| \| Adamtsl4 \| \| Ace \| \| C1qc \| \| Dusp3 \| \| Ccm2l \| \| Bcl2a1d \| \| Klf2 \| \| Kcnt2 \| \| Cdh1 \| \| Eef1b2 \| \| Actl6a \| \| Tmem184b \| \| Ly6c1 \| \| Nos3 \| \| Epb41l4a \| \| Clip1 \| \| Atp1b1 \| \| Sqrdl \| \| Rrm2b \| \| Plac9a \| \| Ptgs1 \| \| Spon1 \| \| Stbd1 \| \| Lamb2 \| \| Rassf8 \| \| Sfxn3 \| \| AI607873 \| \| Lilr4b \| \| St6galnac2 \| \| Myl9 \| \| Cyp1b1 \| \| Gstk1 \| \| Fam107a \| \| Lyz1 \| \| Nme7 \| \| Dbp \| \| Fmo5 \| \| Col4a3 \| \| Cd53 \| \| Arpc3 \| \| Marveld1 \| \| Nrg1 \| \| Cfp \| \| Cdc42ep3 \| \| Smad1 \| \| Parm1 \| \| Kif1b \| \| Mterf4 \| \| Hist2h3b \| \| Btg1 \| \| Fgr \| \| Slc9a3r2 \| \| Cstf3 \| \| Mlec \| \| Adgre1 \| \| Ski \| \| Rcc1 \| \| Aph1b \| \| Klf7 \| \| Klf12 \| \| Cald1 \| \| Nrbp2 \| \| Cotl1 \| \| Fhl1 \| \| S100a9 \| \| Cxcl12 \| \| Hsd3b7 \| \| Mcam \| \| Amigo2 \| \| Birc5 \| \| Stk17b \| \| Car8 \| \| Mrc1 \| \| Nod2 \| \| Cd44 \| \| Gria3 \| \| Vmp1 \| \| Nme1 \| \| Pkib \| \| Lst1 \| \| Col8a1 \| \| Vav1 \| \| Il10ra \| \| Pcolce2 \| \| Lyz2 \| \| Lilrb4a \| \| Mmp13 \| \| Rnf144a \| \| Rnase6 \| \| Klk11 \| \| Rab27a \| \| Klk8 \| \| Marcks \| \| Hdgfrp3 \| \| Mndal \| \| Igf2 \| \| Dhh \| \| Ddah1 \| \| Rgs10 \| \| Epas1 \| \| Itgb4 \| \| Plec \| \| Ecm1 \| \| Cybb \| \| Cdca8 \| \| Klf9 \| \| Galnt15 \| \| Cd52 \| \| Bcl2a1b \| \| Fam129b \| \| Rpl24 \| \| Hist1h3h \| \| Cldn15 \| \| Cd300ld3 \| \| Krt80 \| \| Prr33 \| \| Cyfip2 \| \| Actb \| \| Ugt1a10 \| \| Ccl4 \| \| Prc1 \| \| Nuak1 \| \| Cmip \| \| Lsr \| \| Cc2d2a \| \| Spint1 \| \| Gng2 \| \| Peg13 \| \| Slc43a2 \| \| Psat1 \| \| Sgms1 \| \| Tubb6 \| \| Gas6 \| \| Ptprc \| \| Plbd1 \| \| Irf5 \| \| Cers6 \| \| Ppic \| \| Lpl \| \| Nrros \| \| Pi16 \| \| Tns2 \| \| Lcp1 \| \| Hba-a1 \| \| Hist1h4a \| \| Bmx \| \| Appl2 \| \| Meis2 \| \| Gja1 \| \| Gmfg \| \| Zwint \| \| Itgb2 \| \| Cxcr4 \| \| Hist1h3e \| \| Maml2 \| \| AB124611 \| \| Fn1 \| \| Sos2 \| \| Il1b \| \| Klra2 \| \| Rassf4 \| \| Aurka \| \| Fyb \| \| Itgax \| \| Tsc22d1 \| \| Myrip \| \| Sla \| \| Uhrf1 \| \| Lgals1 \| \| Fam105a \| \| Hist1h4c \| \| Tubb2b \| \| Cgnl1 \| \| Hist1h3d \| \| Il17ra \| \| Hist2h2ab \| \| Rab7b \| \| C3ar1 \| \| Mxi1 \| \| Pacs1 \| \| Cytl1 \| \| Was \| \| Apobr \| \| Itpr1 \| \| Wasf2 \| \| Mfsd6 \| \| Ankle1 \| \| Siglece \| \| Adgre4 \| \| Lgals3 \| \| Lamb1 \| \| Lcp2 \| \| Ndn \| \| Adh7 \| \| Hist1h4j \| \| Gpr65 \| \| Fam49a \| \| Hist2h2ac \| \| Cks1b \| \| Rnd3 \| \| Ypel3 \| \| Hist1h2an \| \| Hist1h2af \| \| Hbegf \| \| Hist2h3c1 \| \| Hist1h4f \| \| Pld4 \| \| Hist1h2ai \| \| Pf4 \| \| Hist1h2ap \| \| Gcnt2 \| \| Clec4a1 \| \| Atp2a3 \| \| Ly6a \| \| Peg3 \| \| AU021092 \| \| Gm2a \| \| Cd300a \| \| Hist1h2ak \| \| Perp \| \| Hist4h4 \| \| Dnah11 \| \| Hist1h2ad \| \| Sod3 \| \| Sult1a1 \| \| S100a4 \| |

**Table 3 GO（BP）enrichment analysis.**

| GO term | Count | PValue |
| --- | --- | --- |
| \| GO:0002376~immune system process \| \| --- \| \| GO:0032776~DNA methylation on cytosine \| \| GO:0007155~cell adhesion \| \| GO:0006335~DNA replication-dependent nucleosome assembly \| \| GO:0045815~positive regulation of gene expression, epigenetic \| \| GO:0051290~protein heterotetramerization \| \| GO:0045766~positive regulation of angiogenesis \| \| GO:0006334~nucleosome assembly \| \| GO:0000183~chromatin silencing at rDNA \| \| GO:0006954~inflammatory response \| | \| 31 \| \| --- \| \| 11 \| \| 32 \| \| 10 \| \| 10 \| \| 10 \| \| 14 \| \| 13 \| \| 10 \| \| 22 \| | \| 4.74E-11 \| \| --- \| \| 2.23E-10 \| \| 3.35E-09 \| \| 7.03E-09 \| \| 7.03E-09 \| \| 1.97E-07 \| \| 4.78E-07 \| \| 6.09E-07 \| \| 8.04E-07 \| \| 2.57E-06 \| |

**Table 4 GO (CC) enrichment analysis.**

| GO term | Count | P Value |
| --- | --- | --- |
| \| GO:0070062~extracellular exosome \| \| --- \| \| GO:0000786~nucleosome \| \| GO:0031012~extracellular matrix \| \| GO:0016020~membrane \| \| GO:0000228~nuclear chromosome \| \| GO:0005615~extracellular space \| \| GO:0005578~proteinaceous extracellular matrix \| \| GO:0009986~cell surface \| \| GO:0005576~extracellular region \| \| GO:0005925~focal adhesion \| | \| \| 123 \| \| --- \| \| 19 \| \| 27 \| \| 180 \| \| 11 \| \| 57 \| \| 21 \| \| 31 \| \| 60 \| \| 23 \| \| \| --- \| --- \| --- \| --- \| --- \| --- \| --- \| --- \| --- \| --- \| --- \| | \| 1.56E-23 \| \| --- \| \| 9.93E-13 \| \| 3.38E-11 \| \| 1.24E-08 \| \| 3.44E-08 \| \| 2.26E-07 \| \| 1.55E-06 \| \| 1.71E-06 \| \| 2.84E-06 \| \| 3.24E-06 \| |

**Table 5 GO (MF) enrichment analysis.**

| GO term | Count | P Value |
| --- | --- | --- |
| \| GO:0005515~protein binding \| \| --- \| \| GO:0042393~histone binding \| \| GO:0031492~nucleosomal DNA binding \| \| GO:0019901~protein kinase binding \| \| GO:0043236~laminin binding \| \| GO:0046982~protein heterodimerization activity \| \| GO:0003779~actin binding \| \| GO:0019899~enzyme binding \| \| GO:0030246~carbohydrate binding \| \| GO:0031720~haptoglobin binding \| | \| \| 123 \| \| --- \| \| 14 \| \| 8 \| \| 22 \| \| 6 \| \| 24 \| \| 18 \| \| 19 \| \| 13 \| \| 3 \| \| \| --- \| --- \| --- \| --- \| --- \| --- \| --- \| --- \| --- \| --- \| --- \| | \| \| 1.23E-08 \| \| --- \| \| 6.10E-07 \| \| 1.01E-05 \| \| 7.84E-05 \| \| 1.08E-04 \| \| 1.18E-04 \| \| 2.39E-04 \| \| 3.74E-04 \| \| 0.001373 \| \| 0.002062 \| \| \| --- \| --- \| --- \| --- \| --- \| --- \| --- \| --- \| --- \| --- \| --- \| |

**Table 6 KEGG pathway enrichment analysis.**

| KEGG term | Count | P Value |
| --- | --- | --- |
| \| mmu05322:Systemic lupus erythematosus \| \| --- \| \| mmu05034:Alcoholism \| \| mmu05150:Staphylococcus aureus infection \| \| mmu05152:Tuberculosis \| \| mmu04145:Phagosome \| \| mmu05144:Malaria \| \| mmu04514:Cell adhesion molecules (CAMs) \| \| mmu05310:Asthma \| \| mmu04672:Intestinal immune network for IgA production \| \| mmu04810:Regulation of actin cytoskeleton \| | \| \| 29 \| \| --- \| \| 23 \| \| 10 \| \| 17 \| \| 15 \| \| 8 \| \| 14 \| \| 6 \| \| 7 \| \| 15 \| \| \| --- \| --- \| --- \| --- \| --- \| --- \| --- \| --- \| --- \| --- \| --- \| | \| \| \| 1.34E-17 \| \| --- \| \| 4.74E-09 \| \| 3.23E-06 \| \| 7.38E-06 \| \| 8.84E-05 \| \| 1.68E-04 \| \| 1.94E-04 \| \| 2.66E-04 \| \| 5.55E-04 \| \| 8.57E-04 \| \| \| --- \| --- \| --- \| --- \| --- \| --- \| --- \| --- \| --- \| --- \| --- \| \| \| --- \| --- \| --- \| --- \| --- \| --- \| --- \| --- \| --- \| --- \| --- \| --- \| |

**Figure S1. Protein–protein interaction networks of differentially expressed genes.**


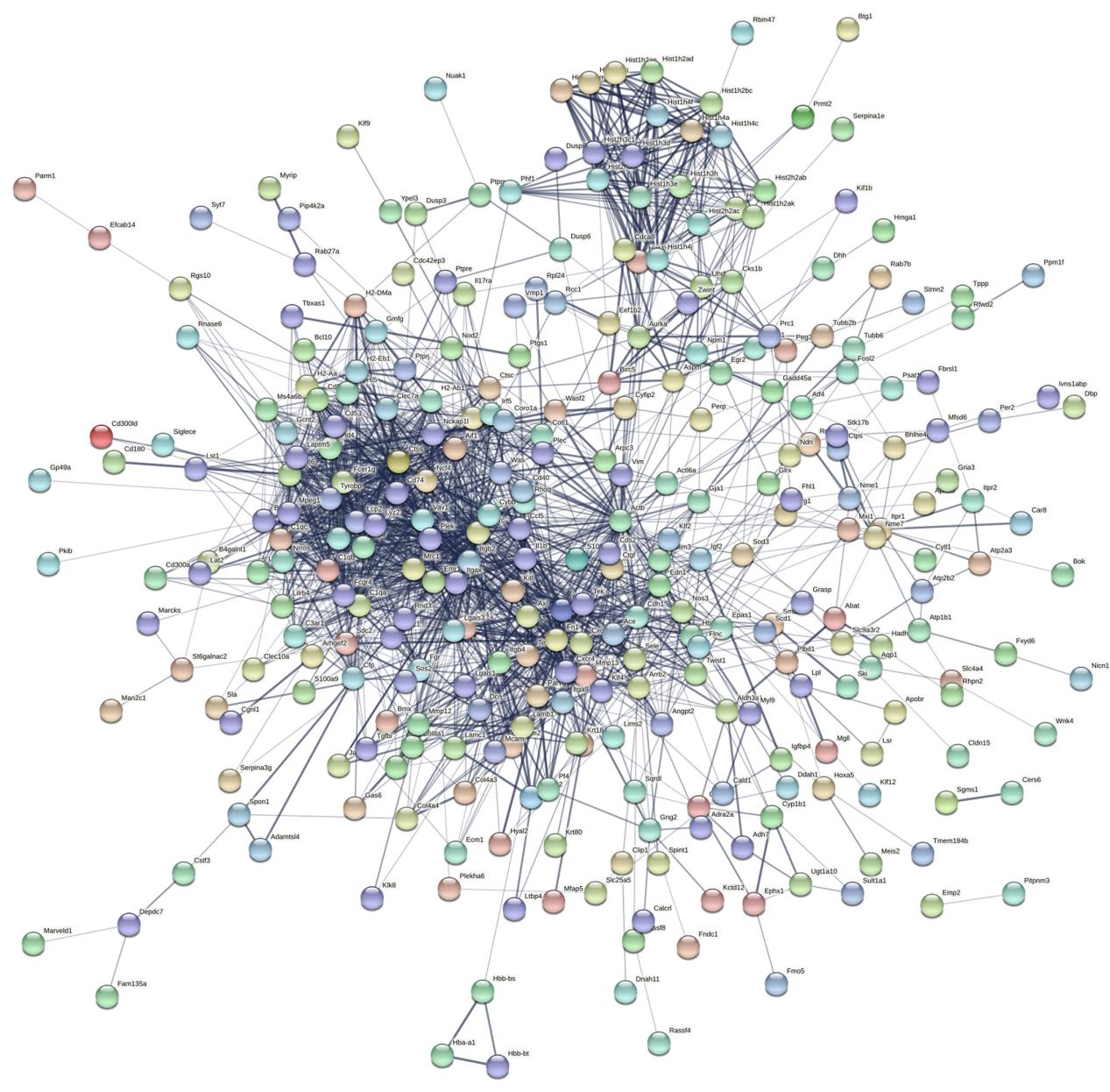


Circles represent genes, lines represent interactions between geneencoded proteins and line colors represent evidence of interactions between proteins.

**Figure S2 Expression of F4/80 protein in the carotid artery 7 days after ligation by immunohistochemistry.**


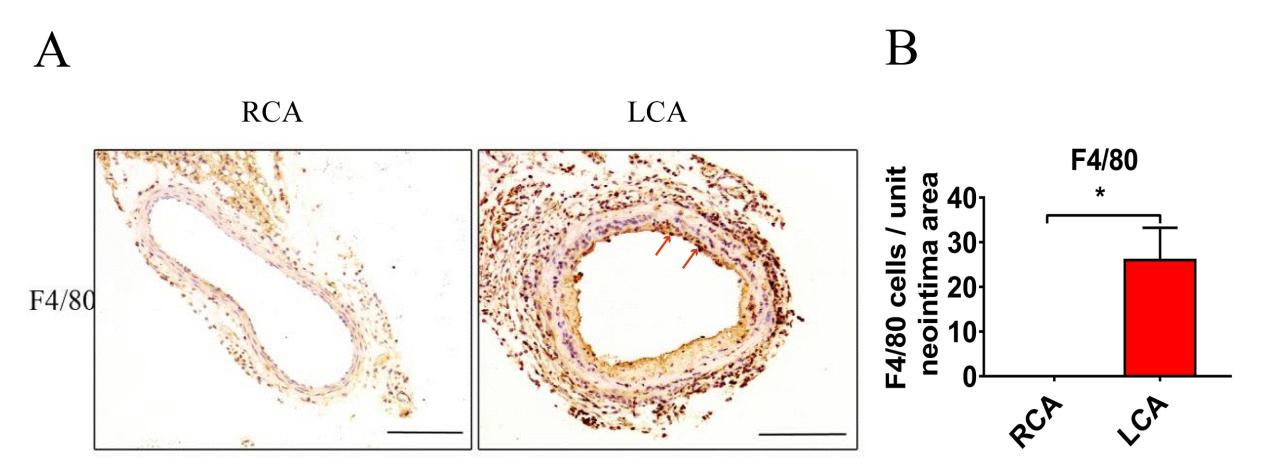


1. **B)** Immunohistochemistry staining and quantitative analysis of F4/80 protein of carotid artery tissues. The arteries were harvested from uninjured RCA (that underwent a sham operation) and injured LCA at 7 days after surgery. The red arrows represent positive cells. Two-tailed unpaired Student’s t-test is used to compare two groups. Data are expressed as means ±SEM. n=3. *, *P*＜0.05; compared with the RCA group. Original magnification, 100x. Scale bar: 50 μm.

**Figure S3 Changes in the expression of BRCC36 mRNA over time.**

**
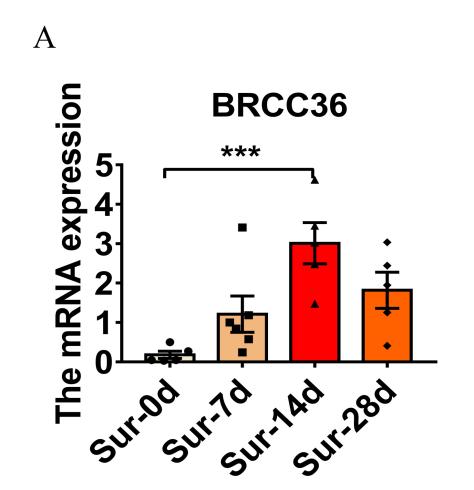
**

The expression changes of BRCC36 mRNA levels in mouse carotid arteries at 0 days, 7 days, 14 days and 28 days after ligation. All values have been standardized by GAPDH. Two-tailed unpaired Student’s t-test is used to compare two groups. Data are expressed as means ±SEM. n=5-6. ***, *P*＜0.001.
